# Occupational exposure to tris (chloropropyl) phosphate in flexible polyurethane foam workers: exposure levels and risk assessment

**DOI:** 10.1093/annweh/wxaf090

**Published:** 2026-01-21

**Authors:** Fatima den Ouden, Patrick de Kort, Yu Ait Bamai, Giulia Poma, Adrian Covaci

**Affiliations:** Toxicological Centre, University of Antwerp, Universiteitsplein 1, Wilrijk 2610, Belgium; EUROPUR AISBL, Avenue de Cortenbergh 71, Brussels 1000, Belgium; Toxicological Centre, University of Antwerp, Universiteitsplein 1, Wilrijk 2610, Belgium; Center for Environmental and Health Sciences, Hokkaido University, Kita 12, Nishi 7, Kita-ku, Sapporo 060-0812, Japan; Toxicological Centre, University of Antwerp, Universiteitsplein 1, Wilrijk 2610, Belgium; Toxicological Centre, University of Antwerp, Universiteitsplein 1, Wilrijk 2610, Belgium

**Keywords:** organophosphate ester flame retardants, urinary biomarkers, occupational exposure, exposure assessment, derived no-effect levels, health-based guidance values

## Abstract

**Introduction:**

Tris (chloropropyl) phosphate (TCPP) is an organophosphate flame retardant (PFR) added to flexible polyurethane foam to adhere to national or sectorial flammability requirements. During the manufacturing of flexible polyurethane foam, workers can be potentially exposed to TCPP dermally or through inhalation.

**Objective:**

We aimed to determine the exposure to TCPP in European polyurethane foam workers and perform a risk assessment based on a newly determined derived no effect level (DNEL).

**Methods:**

In this study, 28 workers from 5 European flexible polyurethane foam production factories participated. Levels of the urinary metabolites of TCPP: bis(1-chloro-isopropyl) hydroxy-isopropyl phosphate (BCIPHIPP) and bis(1-chloro-isopropyl) phosphate (BCIPP) were measured using LC-MS/MS and risk assessment was performed by calculating estimated daily intakes (EDIs) of TCPP and comparing these with the DNEL.

**Results:**

BCIPHIPP was detected in 100% of samples, with a median of 5.56 ng/mL (maximum 420 ng/mL). BCIPP had an overall detection frequency of 31%, but in workers from one individual factory it was detected in 93% (*n* = 13) of samples, with a median of 9.41 ng/mL (maximum 58.6 ng/mL). A DNEL of 3.0 mg/kg bw/day for TCPP was determined for an occupationally exposed population by evaluating a recently published chronic in vivo study on TCPP carcinogenicity. EDIs were all more than 2 orders of magnitude below the DNEL.

**Conclusions:**

Although BCIPHIPP levels detected in this study were higher than in the general population, the performed risk assessment indicated that the included workers are not expected to be at risk for carcinogenic effects following TCPP exposure at the measured levels and that the applied safety measures are sufficiently protecting the workers under these conditions.

What's Important About This Paper? This study reports concentrations of tris (chloropropyl) phosphate (TCPP) metabolites in workers in flexible polyurethane foam production sites in Europe. A derived no-effect level (DNEL) for TCPP was derived from a newly available 2-yr toxicological study in mice and rats, and estimated daily intakes among participants were below the DNEL, suggesting low risk of adverse health effects under the measured conditions. To our knowledge, this is the first study looking into TCPP exposure in flexible polyurethane foam workers.

## Introduction

Flame retardants are added to textiles, plastics, furniture, electronic devices, and other consumer goods to adhere to flammability standards, which are set to reduce the risk of fire. Organophosphate flame retardants (PFRs) are often proposed as alternatives since the ban on certain brominated flame retardants (BFRs) (van der Veen and de Boer 2012). The European Chemicals Agency (ECHA) reported that 18% of the worldwide FR comprises PFRs (European Chemicals Agency 2023a).

Tris (chloropropyl) phosphate (TCPP) is one of the major PFRs and is mainly used globally in polyurethane applications (EU RAR, 2008; Brandsma et al. 2021), predominantly in the production of either foamed rigid PU insulation panels or combustion modified flexible polyurethane foam. Within the United States, spray foam is another significant use, whereby a liquid polyurethane system is sprayed by professionals on construction sites. This is a relatively small use within the EU. The production processes of different types of polyurethanes are described in Brereton et al. (2019). TCPP is used in these applications because of its high efficacy in achieving flame retardancy of the polyurethane material, low cost, and its limited adverse impact on the mechanical and physical properties of the foam compared to other flame retardants (Cooper et al. 2016; European Chemicals Agency 2018; European Chemical Agency 2023a). Commercial TCPP is a mixture of 4 isomers: tris(1-chloro-isopropyl) phosphate (TCIPP; 50 to 85%), bis(1-chloro-isopropyl) 2-chloropropyl phosphate (15 to 40%), bis(2-chloropropyl) 1-chloro-isopropyl phosphate (<15%), and tris(2-chloropropyl) phosphate (<1%) (The Danish Environmental Protection Agency 2014; Truong et al. 2017; European Chemicals Agency 2018; Collins et al. 2022; European Chemicals Agency 2023b). For a more extensive discussion and a proposed harmonized naming convention for TCPP and its metabolites, see Text S1.

In 2008, a European Union Risk Assessment report concluded, based on a qualitative read-across, that due to the chemical structural similarity of TCPP to tris (chloroethyl) phosphate (TCEP) and tris (1,3-dichloro-2-propyl) phosphate (TDCPP), TCPP might also cause carcinogenic adverse effects, but that the weight of evidence was insufficient for classification (EU RAR, 2008). Later, a 2018 screening report by ECHA identified a possible risk for children from exposure to TCPP in flexible polyurethane foams in childcare articles and residential upholstered furniture (European Chemicals Agency 2018). In addition, the National Toxicology Program (NTP) of the United States recently conducted a long-term carcinogenic study in mice and rats and found “some evidence’ for carcinogenic effects of TCPP in male and female rats and male mice, while in female mice “clear evidence’ was concluded (NTP, 2023). This has led to the self-classification of TCPP as a Carcinogen Class 2 (suspected human carcinogens) by REACH Registrants (European Chemicals Agency 2024) following UN GHS criteria (United Nations 2023). Human epidemiological studies showed associations between exposure to TCPP of the general population and development of asthma and allergies (Araki et al. 2014; Araki et al. 2018) and the development of gestational diabetes (Deng et al. 2024). TCPP is a PFR with a higher volatility than some other PFRs, such as TDCPP or newer (proprietary) alkyl phosphate oligomers, and is therefore more likely to be emitted from materials, leading to a higher potential for human exposure through inhalation of contaminated air (EU RAR, 2008; European Chemicals Agency 2018; Estill et al. 2019).

Occupational exposure to TCPP can occur through inhalation or dermal contact during primary production of TCPP, use in an industrial setting (eg production of rigid or flexible foam), handling of articles containing TCPP (eg conversion of combustion modified flexible polyurethane foam), professional use of mixtures containing TCPP (eg spray foam operators), or the waste stage (eg carpet workers removing old carpet underlay) (Brereton et al. 2019; Estill et al. 2020; Shi et al. 2020; Estill et al. 2024).

Estill et al. (2020) detected TCPP in 100% of air samples (particulate phase) and hand wipes taken from 11 workers involved in foam production in the United States. While it was not specified whether flexible or rigid foam was produced, the description of the workers' task implies that rigid foam was being produced. Air and hand wipe concentrations of TCPP were higher in the foam manufacturing industry than in the other workplaces included in the study (eg carpet installation, roofing, gymnastics), except for the spray foam industry which showed higher concentrations in hand wipes than foam production. In addition, bis(1-chloro-isopropyl) phosphate (BCIPP), a metabolite of TCPP, was found in the collected urine samples from foam manufacturing workers with a 100% detection frequency (DF) (Estill et al. 2024). Apart from this study, no other studies have been done specifically on TCPP exposure in rigid foam manufacturing workers. However, Estill et al. (2019) conducted a study in spray foam workers and detected TCPP in all hand wipes of workers with significantly higher TCPP concentrations post-shift than pre-shift (Estill et al. 2019). In the same study, BCIPP was detected in all urine samples of workers with post-shift concentrations being significantly higher than pre-shift concentrations (Estill et al. 2019). Bello et al. (2018) collected air samples, gloves and urine from spray foam workers and foam removers and detected TCPP in 100% of the air samples and gloves. In addition, 2 TCPP metabolites, BCIPP and bis(1-chloro-isopropyl) 1-hydroxy-isopropyl phosphate (BCIPHIPP), were detected in all collected urine samples with post-shift concentrations significantly higher than pre-shift concentrations. To our knowledge, these are the only studies conducted in workers producing or applying rigid polyurethane foam, and they were all conducted in the United States. No studies investigating exposure during flexible polyurethane foam production currently exist.

Urinary measurements of suitable biomarkers are helpful in assessing TCPP exposure, since they reflect multiple exposure pathways. Therefore, in this study, we aimed to determine the concentrations of 2 TCPP metabolites, BCIPP and BCIPHIPP, in urine from workers in the European flexible polyurethane foam manufacturing industry and to estimate their risk of TCPP exposure. In addition, we reviewed the recently published data by the NTP program regarding an extensive chronic in vivo study on the long-term toxicity in rats and mice to determine a derived no-effect level for TCPP (NTP, 2023). The health risk from TCPP exposure in workers was assessed by calculating estimated daily intakes (EDIs) of workers and comparing these values with the derived DNEL. This work can be helpful in assessing the exposure and risk to TCPP in a highly exposed population and clarifying the need for measures to reduce their TCPP exposure.

## Materials and methods

Details about chemicals and reagents can be found in Text S2.

### Study population and sample collection

Flexible polyurethane foam production factories that produce combustion modified foam and are affiliated with EUROPUR, the European association of flexible polyurethane foam blocks manufacturers, were approached to participate in this study. In total, 5 factories across Europe agreed to participate, resulting in the collection of 65 urine samples. Of these factories, 4 factories were located on the European mainland, while one was in the United Kingdom and Ireland region. In the United Kingdom and Ireland, stricter fire safety requirements for foam filling are in place requiring foam filling used in domestic furniture and mattresses to be able to resist the CRIB-5 energy source (UK Statutory Instruments 1988; The Danish Environmental Protection Agency 2016; European Chemicals Agency 2018). Of the collected samples, 42 were taken from 21 foam line workers (21 pre- and 21 post-shift samples), 14 were taken from 7 conversion workers (7 pre- and 7 post-shift samples), and 9 were control samples (Fig. S1 and Table S1). From factories C002 and C006, only samples from foam line workers were received, while factories C007, C012, and C013 included both foam line and conversion workers. A description of the foam production process, the detailed activities of foam line workers and conversion workers, and applied risk management measures can be found in Text S3.

Foam workers include workers who are involved in the production of foam and are present when TCPP is added to the mixture of raw materials during foam production. Conversion workers are involved in the processing of foam blocks, after the foam production, eg cutting them into the desired size and shape. Control samples included participants that worked on the factory grounds but were not directly involved in flexible foam production (eg administrative workers). Workers from participating factories were informed about the aim of the study, that result may be published, and about what would be done with their urine samples. It was emphasized that participation in the study was voluntary and that refusal would not have any consequences for their employment. Workers who agreed to participate gave informed consent and were asked to deliver 2 spot urine samples, one sample before the start of their shift, the second sample after finishing their shift. In addition, data on sex and type of work (working at the foam tunnel or working in the conversion of foam blocks) were collected. Samples were collected in plastic tubes or cups and sent to the analyzing lab. Samples were stored at −20 °C until analysis. This study involved the non-invasive collection of urine samples and workers were not exposed to an additional risk by participating in this study. In terms of data protection, a personal data spread minimization policy was followed: participating factories provided only anonymized information along with the samples submitted (sex and type of work) and only PdK knows the identity of the participating factories. As such, the authors were dealing only with deidentified anonymized samples. For the above-mentioned reasons, ethical approval was not sought (World Medical Association 2001; Hrynaszkiewicz et al. 2010; Regulation 536/2014).

### Analysis of PFR metabolites in urine

To determine urinary concentrations of BCIPP and BCIPHIPP, urine samples were extracted using solid-phase extraction and analyzed using liquid chromatography coupled to mass spectrometry (LC-MS/MS) following a previously validated method (Bastiaensen et al. 2018). A description of the extraction procedure and analytical methods is reported in Text S4. Details of the analytical method were reported in Bastiaensen et al. (2018), while adapted parameters for analysis on the triple quadrupole mass spectrometer ESI-6495 are shown in Tables S2 and S3.

To obtain more information on the kinetic behavior of TCPP, and more specifically if BCIPHIPP is excreted primarily as BCIPHIPP itself or as BCIPHIPP glucuronic acid conjugate, 4 urine samples were re-extracted with and without adding β-glucuronidase enzyme. All samples were extracted in duplicate for each condition.

### Quality control

BCIPHIPP and BCIPP were quantified using linear calibration curves consisting of 9 calibration points ranging from 0.04 to 100 ng/mL for BCIPHIPP and 0.2 to 250 ng/mL for BCIPP. The limit of quantification (LOQ) was 0.04 ng/mL for BCIPHIPP and 1.00 ng/mL for BCIPP. To avoid contamination, glassware was rinsed with acetone and baked overnight at 300 °C. In addition, the sample preparation was performed in a precleaned flow cabinet. A procedural blank was included in each batch (*n* = 21 samples, 3 blanks in total) and analyzed to determine background contamination. A quality control (QC) was included in each batch (*n* = 21 samples, 3 QCs in total) by spiking urine with 50 ng BCIPP and 10 ng BCIPHIPP. An unspiked urine sample was included to correct for levels already present in urine. Levels detected in the blank samples were < LOQ for both BCIPP and BCIPHIPP, while accuracies in spiked urine were 94% and 62%, respectively.

### Data analysis

Specific gravity (SG) of each urine sample was measured using a handheld refractometer (Euromex Rf.6612 Euromex Arnhem, the Netherlands) to correct for the differences in urine dilution between samples. The following formula was used to correct for the specific gravity:


$$conc_{SG}=conc\;*\;\frac{1.024-1}{SG-1}$$

In this formula, *conc_SG_* is the metabolite concentration corrected for SG in ng/mL, *conc* is the found metabolite concentration in the urine sample in ng/mL and *SG* is the measured specific gravity (Pearson et al. 2009; Kuiper et al. 2021). Specific gravity corrected concentrations were used for further data analysis and the concentrations reported in the main text are specific gravity adjusted concentrations. Figures were made using Graphpad version 10.6.1.

### Statistical analysis

All statistical analyses were performed for compounds with DF > 60% and the values below LOQ were replaced by LOQ*DF. Since concentrations of BCIPHIPP and BCIPP were not normally distributed, non-parametric statistical tests were performed. Statistical analyses were performed by considering samples from workers and controls collected from all participating factories.

A Mann–Whitney test was performed to examine the differences between control versus pre-shift and between control versus post-shift. Since pre-shift and post-shift samples came from the same individual, the Wilcoxon matched paired test was used to examine the differences between pre-shift and post-shift concentrations. Workers could also be divided into workers involved in foam production and workers involved in the conversion of foam. To test for differences between types of workers (controls, foam line workers, and conversion workers), a Kruskal–Wallis test followed by a multiple comparison Dunn's test set control workers as “control” was performed. For this test, the pre- and post-shift samples from the foam line and conversion workers were considered as separate groups. A 2-tailed test and a 5% level of significance were used for all analyses. For multiple comparisons between control, pre-shift, and post-shift samples, the Bonferroni correction (*P* < 0.05/n) was applied. Statistical analyses were performed using SPSS version 28.0.1.1 and Graphpad version 10.6.1.

### Risk assessment

To assess the risk of TCPP exposure in occupationally exposed workers, EDIs for TCPP were calculated using the following equation (Chen et al. 2018):


$$EDI=\left(\frac{c_{meta}\;*\;V_{urine}}{F_{UE}\;*\;bw}\right)\;*\;\frac{MW_{p}}{MW_{m}}$$

In this formula, EDI is the estimated daily intake in ng/kg bw/day, *c_meta_* represents the metabolite concentration in ng/mL (corrected for specific gravity), *V_urine_* is the daily excreted volume of urine which was estimated at 1,600 mL/day for adult males and 1,200 mL/day for adult females (Valentin and Streffer 2002), *F_UE_* is the urinary excretion factor, *bw* is the body weight in kg for which 70 kg was assumed for adult male and female workers (European Chemicals Agency 2012), and *MW_p_* and *MW_m_* are the molecular weight of the parent compound and the metabolite in g/mol, respectively.

While studies on the toxicokinetics of TCPP are scarce, a F_UE_ value was extracted based on available toxicokinetic data from literature describing in vitro experiments and rat studies (discussed in section Toxicokinetic behavior of TCPP and Text S6). Obtained EDI values were divided by the DNEL to obtain a risk characterization ratio (RCR) and assess the potential risk for workers following exposure to TCPP. Multiple DNEL values for TCPP have already been reported in literature (EU RAR, 2008; Van den Eede et al. 2011; Ali et al. 2012; United States Environmental Protection Agency 2012; European Chemicals Agency 2018; Poma et al. 2019; Zhao et al. 2019). However, the NTP has recently published an extensive chronic in vivo study on the long-term toxicity of TCPP in rats and mice (NTP, 2023). Data from this report were reviewed in the present study and used to derive a DNEL value for TCPP (see section Derivation of a DNEL for TCPP and Text S5-2).

## Results and discussion

### Levels of TCPP metabolites in workers

BCIPHIPP was detected in all 65 samples, ranging from 0.70 to 420 ng/mL with a median of 5.56 ng/mL (Table 1). The highest median BCIPHIPP concentration was found in factory C007 (85.4 ng/mL, Table 2). BCIPP was detected in 20 of the 65 samples (DF = 31%) overall, but in factory C007, this compound was detected in 13 out of 14 samples (93% DF), with a median level of 9.41 ng/mL (Table 1, Table 2). A possible reason for the lower DF of BCIPP might be because the LOQ of this compound is 25 times higher than the LOQ for BCIPHIPP.

**Table 1:** Distribution of BCIPHIPP and BCIPP concentrations (ng/mL, SG corrected), EDIs (ng/kg bw/day) and RCRs in polyurethane foam factory workers and control samples (*n* = 65).

	LOQ	% >LOQ	Min	25th percentile	Median	75th percentile	Max
BCIPHIPP (ng/mL)	0.04	100	0.70	2.51	5.56	16.0	420
BCIPP (ng/mL)	1.00	31	<LOQ	<LOQ	<LOQ	1.23	58.6
EDI TCPP							
(ng/kg bw/day)	NA	NA	34	123	270	750	20,342
RCR TCPP	NA	NA	1.1*10^−5^	4.1*10^−5^	9.0*10^−5^	2.5*10^−4^	6.8*10^−3^

**Table 2:** Median concentrations and interquartile ranges for BCIPP and BCIPHIPP (ng/mL, SG-corrected) in the different factories.

	Factories					
	C002 (*n* = 14)	C006 (*n* = 6)	C007 (*n* = 14)	C012 (*n* = 20)	C013 (*n* = 11)	Overall
BCIPHIPP (ng/mL)	1.90 (1.05 to 3.03)	7.72 (6.06 to 9.96)	85.4 (49.4 to 284)	3.81 (2.68 to 5.55)	11.5 (2.49 to 16.5)	5.56 (2.51 to 16.0)
BCIPP (ng/mL)	<LOQ	<LOQ (<LOQ-0.91)	9.41 (4.67 to 31.3)	<LOQ	<LOQ (<LOQ-1.24)	<LOQ (<LOQ-1.23)

**Figure 1:**
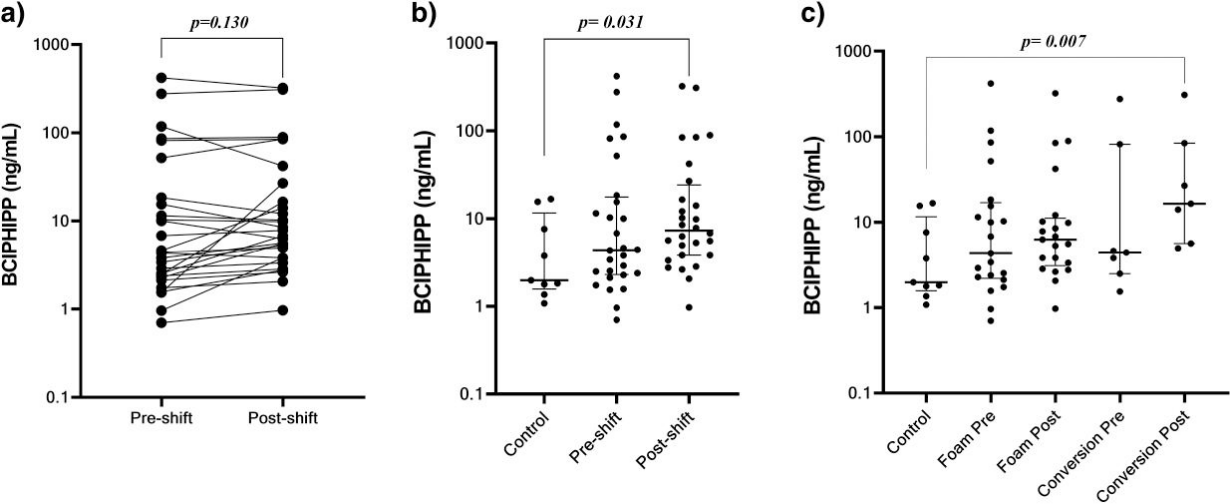
Comparison of BCIPHIPP concentrations (SG corrected) between pre- and post-shift workers (a, Wilcoxon matched paired test), comparison of BCIPHIPP concentration between controls and pre-shift concentrations and controls and post-shift workers (b, Kruskal-Wallis test followed by Dunn's test) and the comparison between controls and foam line workers (pre-shift and post-shift separately) and controls and conversion workers (pre-shift and post-shift separately) (c, Kruskal–Wallis test followed by Dunn's test). Statistical significances for multiple comparisons (b and c) were considered as *P* < 0.05/*n* (*n*: number of comparisons); b: *P* < 0.017, c: *P* < 0.013.

BCIPHIPP concentrations in pre-shift samples were compared with concentrations in post-shift samples (Fig. 1a). No significant differences could be detected between pre-shift and post-shift levels of BCIPHIPP in the included samples (*P*  *=* 0.130).

In addition, BCIPHIPP concentrations of control workers were compared to pre-shift and post-shift samples (Fig. 1b). Pre-shift and post-shift BCIPHIPP levels were not significantly different from controls (*P* = 0.208 and *P* = 0.031, respectively). When stratifying for the different work activities (foam line workers or conversion workers), post-shift samples from conversion workers showed significantly higher BCIPHIPP concentrations than controls (*P* = 0.007), while for foam line workers (pre-shift and post-shift) and pre-shift samples from conversion workers, no statistically significant differences in BCIPHIPP concentrations were found compared to control samples (Fig. 1c).

As the concentrations in factory C007 were substantially higher than in the other factories, and this could affect the comparison, the statistical analyses were also performed excluding the samples from factory C007. No significant differences were found between the pre-shift and post-shift levels (*P* = 0.094). In addition, no significant differences could be detected between control and pre-shift levels (*P* = 0.151). When excluding factory C007, post-shift levels were significantly higher than the control levels (*P* = 0.006). When considering the different work activities, post-shift samples from conversion workers showed higher BCIPHIPP levels than controls (*P* = 0.001), but no other significant differences were observed. However, the omission of factory C007 from the analyses resulted in a lower sample size in the control group and subgroups used for statistics and, therefore these results should not be overinterpreted.

Median concentrations of both BCIPHIPP and BCIPP in the flexible polyurethane foam workers were higher than those reported in the general European population (Bastiaensen et al. 2019; Xu et al. 2019; van der Schyff et al. 2023). BCIPHIPP concentrations in the current study were lower than reported by Bello et al. (2018) in urine from 24 spray polyurethane foam workers in the United States (mean 88.8 ng/mL, range 5.32 to 703 ng/mL). However, BCIPHIPP concentrations in factory C007 showed similar concentrations (median 85.4 ng/mL, range 49.4 to 284 ng/mL) to those reported by Bello et al. (2018). To our knowledge, the study of Estill et al. (2024) is the only study using urine measurements of BCIPP to assess occupational exposure to TCPP in 11 workers at an industrial production site in the United States; however this was rigid foam, which has a substantially different production process. Estill et al. (2024) detected BCIPP in all samples with a mean pre-shift concentration of 0.73 ng/mL and a mean post-shift concentration of 4.03 ng/mL. Bello et al. (2018) included 24 spray foam workers in the United States and reported a mean BCIPP urine concentration of 6.2 ng/mL (1.2 to 51.4 ng/mL). The BCIPP concentrations and DF in the current study are lower than the concentrations and DFs reported by Estill et al. (2024) and Bello et al. (2018), except for the BCIPP levels measured in factory C007 which are similar. The current study also reports lower BCIPP concentrations compared with Estill et al. (2019), who measured BCIPP in urine from 29 spray foam workers in the United States (median 52 ng/mL, 2.31 to 5,240 ng/mL). Differences in concentrations can be explained by geographical differences and the fact that spray foam workers might be exposed to a higher extent to TCPP than workers in a flexible foam production site (Estill et al. 2024).

The high concentrations of TCPP metabolites found in factory C007 are due to its localization in the United Kingdom and Ireland region, where stricter fire regulations are in place. These stricter requirements can only be met by using a combination of melamine and TCPP, meaning that all upholstered furniture and mattresses produced for this region contain TCPP, which can explain a higher exposure to TCPP in workers. In addition, this requirement leads to an overall higher exposure to flame retardants in this region, which can be seen from the high values in control samples from this factory (*n* = 2, < LOQ and 1.97 ng/mL for BCIPP, 15.6 and 16.7 ng/mL for BCIPHIPP). To our knowledge, no levels of TCPP metabolites in urine were reported for the general population in the United Kingdom. However, Brommer and Harrad (2015) analyzed PFR levels in dust and found TCPP concentrations in different micro-environments in the United Kingdom to be about 10 times higher than TCPP concentrations reported in dust from other European countries, which supports the urinary findings.

We have considered background exposure by obtaining control samples from people working on factory grounds but not in contact with TCPP itself. Since these samples were from different individuals than the workers, it might be possible that (some of) the individual workers have a slightly higher background exposure, and increased concentrations in TCPP metabolites might not be due only to exposure to TCPP in the factory. In addition, it cannot be excluded that pre-shift samples still reflected exposure from the previous workday or that the peak metabolite concentrations due to TCPP exposure during the workday were not yet reached at the time of donating the post-shift sample. Therefore, for similar future work, it is recommended to collect pre-shift samples following a period for which the workers were away from work (eg weekend) and to conduct sampling during several days during the workweek to investigate if accumulation occurs during the workweek.

Another limitation of the study is the rather small sample size of workers and controls from each factory. Therefore, the results of the statistical analysis for subgroups should be interpreted with caution. Additionally, the small sample size might limit the representativeness of other European factories. Ideally, from an academic perspective, more workers and controls should be included to provide more robust statistical and more representative results in future studies. However, it is not always feasible to include more workers, as the number of individuals performing such activities tends to be minimized for economic and safety reasons. To give a practical image, a flexible polyurethane foam slabstock foam line tends to be operated by 3 to 10 foam line operators (normally 6). There are roughly 25 to 30 factories in the European Economic Area (EEA) and UK that produce combustion modified flexible polyurethane foam (ie foam with flame retardants), meaning that the total workforce exposed to TCPP in flexible foam production is around 150 to 180 workers. With samples of 21 foam line workers, already between 12 and 14% of all possible participants in EEA + UK participated. With regards to the number of workers converting TCPP containing foam, the total number of workers is vastly greater. The total number of workers exposed in conversion and handling of TCPP containing foam will likely be measured in the tens of thousands and the 7 conversion workers included in the current study might therefore not be representative for all conversion workers. A way to increase the power of future studies may be best sought in taking more samples for each individual over a period of time.

Finally, as factories participated voluntarily, there might be a selection bias, with factories that correctly implement safety measures being more prone to participate in the study.

### Toxicity of TCPP

The current health-based guidance values (HBGVs) based on toxicological evaluation have been derived from an EU Risk Assessment Report (EU RAR, 2008), Van den Eede et al. (2011), an US EPA report (United States Environmental Protection Agency 2012), and an ECHA Screening Report (European Chemicals Agency 2018). At the time of the derivation of these HBGVs, the highest quality repeated dose toxicity available studies were subchronic study reports. The endpoint identified by all reports as a Point of Departure (PoD) was increased liver weight/hepatocyte hypertrophy, which was assumed to be a prelude to tumorigenesis, with most authority reports concluding that this would be through a threshold mode of action. A detailed description of the studies that derived current HBGVs can be found in Text S5.

#### Derivation of a DNEL for TCPP

The United States NTP Program (US NTP) has recently performed a chronic (2 yr) study in rats and mice to evaluate the carcinogenic potential of TCPP (NTP, 2023). A detailed evaluation of this study and the proposed PoDs are reported in Text S5-1 and Text S5-2. For male rats, the critical endpoint was hepatocellular adenoma or carcinoma with 294 mg/kg bw as PoD, while for female rats, a PoD of 259 mg/kg bw was set based on the endpoint of uterine adenoma or carcinoma. For male mice, no PoD was identified, since no critical endpoints were affected. However, for female mice, a PoD of 329 mg/kg bw was identified based on the occurrence of hepatocellular adenoma or carcinoma.

The PoDs derived from the chronic NTP study were further used to calculate DNELs for TCPP for workers and the general population (Table 3). This was done by correcting for interspecies (factor 10 for rats, factor 17.5 for mice) and intraspecies (factor 5 for workers, factor 10 for the general population) differences (European Chemicals Agency 2012). In addition, a correction factor of 0.8 was applied for the oral absorption (EU RAR, 2008; European Chemicals Agency 2018). No correction was applied for dose–response relationships or exposure duration based on the chronic nature of the study.

**Table 3:** Derived DNEL values based on NOAELs* or BDM_5_** of adverse effects of carcinogenicity in the NTP study.

	Male rats		Female rats		Female mice	
	Hepatocellular adenoma or carcinoma		Uterine adenoma or adenocarcinoma		Hepatocellular adenoma or carcinoma	
	Worker	General population	Worker	General population	Worker	General population
Point of departure (mg/kg bw/day)	294*		259**		329*	
Correction factor interspecies	10		10		17.5	
Correction factor intraspecies	5	10	5	10	5	10
Oral DNEL (mg/kg bw/day)	5.9	2.9	5.2	2.6	3.8	1.9
Correction oral absorption	0.8	0.8	0.8	0.8	0.8	0.8
**Internal DNEL (mg/kg bw/day)**	**4.7**	**2.4**	**4.1**	**2**.**1**	**3**.**0**	**1**.**5**

The most sensitive DNEL was 3.0 mg/kg bw/day for workers and 1.5 mg/kg bw/day for the general population. These DNELs are approximately 2 orders of magnitude higher than previously reported HBGV for TCPP in literature (Table S4). This is because previously reported HBGVs were mostly based on NOAELs, LOAELs or BMDs obtained in subchronic studies that did not always have a very clear dose response curve and were performed with doses that did not allow for the establishment of a NOAEL, but rather a LOAEL (considering such uncertainty necessitated the use of larger assessment factors). This chronic study in 2 species with appropriately selected doses clearly demonstrates the threshold mode of action for the liver effect and allows for the establishment of a clear PoD (NTP, 2023), and for the use of lower assessment factors of 87.5 and 175 (for workers and consumers, respectively) as one needs only correct for inter- and intraspecies differences (European Chemicals Agency 2012). As we have calculated the DNEL based on the NTP study, we cannot make any statements regarding any other adverse effects than carcinogenic effects. However, the NTP study was conducted because it was assumed that carcinogenic effects might be the critical endpoint for TCPP.

### Toxicokinetic behavior of TCPP

To use appropriate kinetic parameters for calculation of EDI values and subsequent risk assessment, available literature on the kinetic behavior of TCPP was evaluated (as detailed in Text S6).

In the evaluated in vivo studies, various oral and intravenous doses resulted in 49% to 77% of the administered TCPP dose being excreted in urine (European Chemicals Agency 1984; Minegishi et al. 1988). Since the occupational exposure will likely be via the inhalation (or dermal) route, which is devoid of first pass metabolism (or first pass excretion in this case), the 63% urinary excretion following intravenous dosing is the most important factor to consider when establishing a urinary excretion factor. Metabolic studies indicate that BCIPHIPP is by far the major metabolite formed and the results of den Ouden et al. (2024) further indicate that the relative abundance of BCIPHIPP, in exposed workers is more than 90%. By extracting samples with and without β-glucuronidase, the current study also established that most of the BCIPHIPP detected in the samples was originally excreted as BCIPHIPP-GlcA in urine (Text S6-Table 2). Therefore, it should be expected that >50%, but less than 63% of the occupational exposure to TCPP, is eliminated as BCIPHIPP and BCIPHIPP-GlcA. To be conservative, a urinary excretion factor (F_ue_) value of 0.5 was used as average excretion factor to perform risk assessment for TCPP exposure.

### Estimated daily intake values for occupationally exposed workers

EDIs were calculated for each worker (both pre-shift and post-shift samples) based on the BCIPHIPP concentrations of individual workers and using the excretion factor (*F*_ue_ = 0.5) derived in the previous section. Calculated EDI values were compared with the DNEL of 3.0 mg/kg bw/day derived in section Derivation of a DNEL for TCPP to assess the risk of workers to the adverse effects of TCPP.

**Figure 2:**
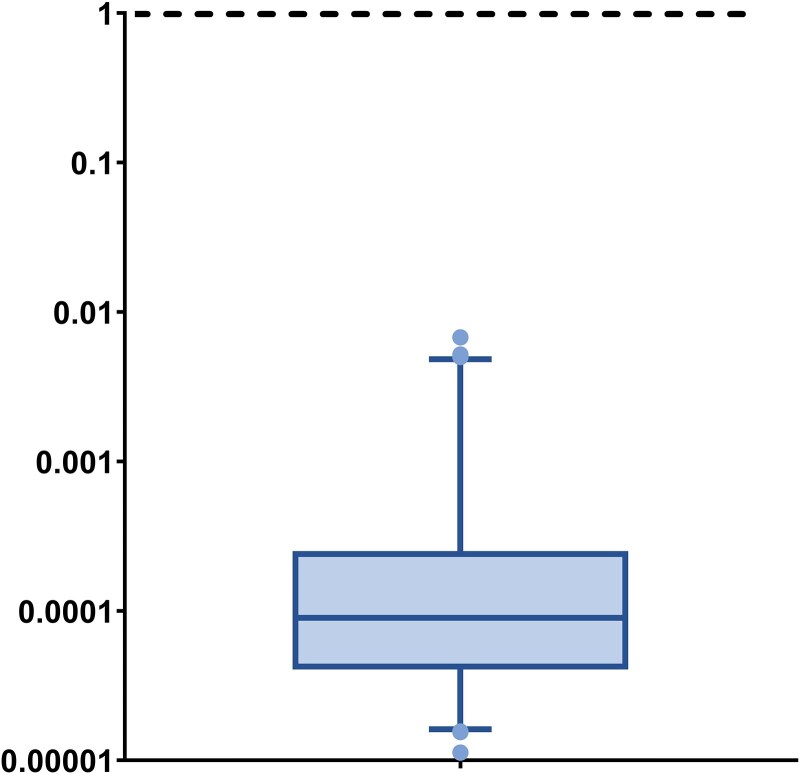
Distribution of RCRs based on BCIPHIPP concentrations in individual samples.

Calculated EDIs ranged from 34 ng/kg bw/day to 20,342 ng/kg bw/day (Table 1) with a median EDI of 270 ng/kg bw/day. Even for the highest calculated EDI, the RCR was still below 0.01 (Fig. 2, Table 1) with only 10 samples (15%) having RCRs above 0.001. These 10 samples were all from individuals working in factory C007. In this factory, which showed the highest BCIPHIPP concentrations, RCR values ranged from 0.0002 in a control sample to 0.007 in a pre-shift sample from a foam line worker. To our knowledge, no EDI calculations based on metabolite concentrations in urine have been done for TCPP in the general population in Europe. In addition, other studies in occupationally exposed workers did not include EDI calculations and risk assessment (Bello et al. 2018; Estill et al. 2019; Estill et al. 2024). The obtained EDI and RCR values, based on the new NTP study, are relatively low compared to the considered DNEL and indicate that flexible polyurethane foam workers included in this study are not at risk of carcinogenic adverse effects of TCPP under the measured conditions. One limitation regarding the assessment of the toxicokinetic behavior of TCPP is that the studies described used oral or intravenous doses of TCPP, while exposure in workers might occur primarily through inhalation and dermal contact with TCPP. This might lead to uncertainty in the estimated daily intake of TCPP for workers. Moreover, the results might not be representative of chronic exposure since samples were only taken at the end of one working day. When interpreting conclusions on risk of exposure, this should be kept in mind.

## Conclusion

To our knowledge, this is the first study to assess exposure to TCPP and relative risks in flexible polyurethane foam manufacturing workers using a new DNEL based on the recently published NTP report. TCPP metabolite levels in polyurethane foam workers were higher than those found in the general population. Compared to other occupational exposure studies, levels of TCPP metabolites found in the current study were similar or lower. However, since no studies on urinary TCPP metabolites in flexible polyurethane foam production factories have been conducted before, it is difficult to make direct comparisons. Based on results of the NTP chronic toxicity study, a DNEL of 3.0 mg/kg bw/day was derived for TCPP which was used to determine the RCR for TCPP in occupationally exposed populations. EDI values were more than 2 orders of magnitude lower than the derived DNEL and therefore it was concluded that workers in flexible polyurethane foam production factories included in this study are not at risk for the carcinogenic adverse effects of TCPP at the measured levels in this study and that implemented safety measures offer sufficient protection under these conditions.

## Supplementary Material

wxaf090_Supplementary_Data

## Data Availability

The full dataset contains limited indirect identifiers (ie sex and occupation), as such this is not available as supplementary information to protect the privacy of the involved workers and participating factories (Hrynaszkiewicz et al. 2010).
