# Supplementary material for: Occupational exposure to tris (chloropropyl) phosphate in flexible polyurethane foam workers: exposure levels and risk assessment
Source: Ann Work Expo Health. 2026 Jan 21;70(1):wxaf090. doi: 10.1093/annweh/wxaf090 (PMC12822784; doi:10.1093/annweh/wxaf090)
Supplement: wxaf090_Supplementary_Data [file wxaf090_supplementary_data.pdf]

Supplementary Information

Occupational exposure to tris (chloropropyl) phosphate in flexible polyurethane foam workers: exposure levels and risk assessment

Fatima den Ouden[1*], Patrick de Kort[2], Yu Ait Bamai[1,3], Giulia Poma[1], Adrian Covaci[1*]

[1] Toxicological Centre, University of Antwerp, Universiteitsplein 1, 2610 Wilrijk, Belgium
[2] EUROPUR AISBL, Avenue de Cortenbergh 71, 1000 Brussels, Belgium
[3] Center for Environmental and Health Sciences, Hokkaido University, Kita 12, Nishi 7, Kita-ku, Sapporo, 060-0812, Japan

*corresponding author: fatima.denouden@uantwerpen.be; adrian.covaci@uantwerpen.be

SI-1 Text
Text S1: Proposed naming convention for TCPP and its metabolites
Text S2:Chemicals and reagents
Text S3: Description of foam production process and activities of foam line workers and conversion workers
Text S4: Quantitative analysis of PFR metabolites in urine
Text S5: Toxicity of TCPP
Text S6: Toxicokinetic behavior of TCPP

SI-2 Tables
Table S1: Overview of included samples
Table S2: Instrumental parameters for BCIPHIPP and BCIPP
Table S3: Instrumental settings for analysis of BCIPHIPP and BCIPP
Table S4: Comparison of HBGVs of TCPP in literature

SI-3 Figures
Figure S1: Overview of study participants

Text S1 Proposed naming convention for TCIPP and its metabolites

Tris(chloropropyl) phosphate (TCPP) is produced by the reaction of phosphoryl trichloride ($POCl_3$) with propylene oxide which is claimed to yield tris(2-chloropropyl) phosphate (Svara et al., 2006). This reaction yields an isomeric mixture with 4 constituent isomers with the following generalized structure:

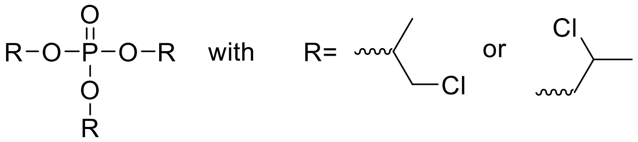

As such REACH Registrants, in discussions with the European Chemicals Agency, have changed the name of their registered substance to: “Reaction products of phosphoryl trichloride and 2-methyloxirane" (S. Kroon, ICL, personal communication, 11 September 2020). The ratio of the 4 isomers is dependent to a degree on the production process, however the “boundary composition” in the registration dossier and described with names for the isomers is included in the US NTP carcinogenicity study report (NTP, 2023) and Danish Substance Evaluation (The Danish Enivronmental Protection Agency, 2014) as shown in Text S1-Table 1. The concentration of the different isomers of a single specific product from one supplier used in the US NTP study is also given as an example.

Text S1-Table 1 Ratio of isomers in TCPP as described by the US NTP and the Danish Environmental Protection Agency

Chemical Name	CAS RN	EC Number	Concentration Range (Commercial Product)	Concentration (Test substance US NTP study)	Other Names
**Tris(1-chloro-2-propyl) phosphate (TCIPP)**	13674-84-5	237-158-7	50-85 %	65-68%	2-Propanol, 1-chloro-, 2,2′,2″-phosphate<br>Tris(2-chloro-1-methylethyl) phosphate<br>Tris(2-chloro isopropyl) phosphate
**Bis(1-chloro-1-methylethyl) 2-chloropropyl phosphate**	76025-08-6	616-283-4	15-40 %	25-27%	Bis(1-chloro-2-propyl) 2-chloro-1-propyl phosphate<br>Bis(1-chloro isopropyl) 2-chloropropyl phosphate

Chemical Name	CAS RN	EC Number	Concentration Range (Commercial Product)	Concentration (Test substance US NTP study)	Other Names
**Bis(2-chloropropyl) 1-chloroisopropyl phosphate**	76649-15-5	616-366-5	<15 %	4%	2-Chloro-1-methylethyl bis(2-chloropropyl) phosphate<br>Bis(2-chloropropyl) 2- chloro-1-methylethyl phosphate<br>Bis(2-chloro-1-propyl) 1-chloro-2-propyl phosphate
**Tris(2-chloropropyl) phosphate**	6145-73-9	228-150-4	<1 %	0.2%	1-Propanol, 2-chloro-, phosphate (3:1)<br>Tris(2-chloro-1-propyl) phosphate

It is clearly observable that the naming convention used is at least inconsistent and even sometimes confusing (Truong et al., 2017). The following naming is hereby proposed and will be maintained throughout the paper:

Text S1-Table 2 Naming of tris(chloropropyl) phosphate and its metabolites used throughout this paper

Structure	Name	Abbreviation	Concentration Range in Commercial TCPP
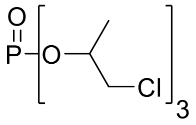	Tris(1-chloroisopropyl) Phosphate	TCIPP	50-85%
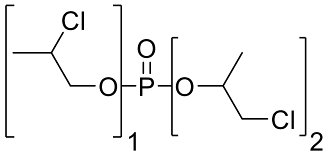	Bis(1-chloroisopropyl) 2-chloropropyl Phosphate	-	15-40%
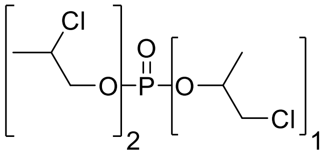	Bis(2-chloropropyl) 1-chloroisopropyl Phosphate	-	<15%
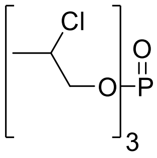	Tris(2-chloropropyl) Phosphate	-	<1%

Various analytical standards may be available and their correct identification in method sections of scientific papers is useful for the interpretation of results. For example, work performed by Den Ouden

et al. (2024) on *in vitro* biotransformation used pure TCIPP, while similar work by Van den Eede et al. (2013) used a “standard for TCIPP (mixture of 3 isomers)”, which is likely commercial TCPP with TCIPP as a main constituent.

As can be seen Text S1-Table 3, in several research papers (Abdallah et al., 2015; den Ouden et al., 2024; Van den Eede et al., 2013, 2015, 2016), metabolites of TCPP have been identified and were given various inconsistent names and abbreviations.

Text S1-Table 3 Naming and abbreviations of TCPP metabolites used in various papers.

Structure	Van den Eede et al. (2013)	Abdallah et al. (2015)	Van den Eede et al. (2015)	Van den Eede et al. (2016)	Den Ouden et al. (2024)
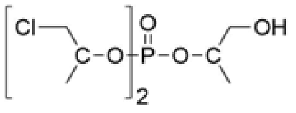	TCIPP-M1	bis(1-chloro-2-propyl) hydroxy 2-propyl phosphate | TCIPP-M1	bis(1-chloro-2-propyl) 1-hydroxy-2-propyl phosphate | BCIPHIPP	bis(1-chloro-2-propyl) 1-hydroxy 2-propyl phosphate | BCIPHIPP	1-hydroxy-2-propyl bis (1-chloro-2-propyl) phosphate | BCIPHIPP
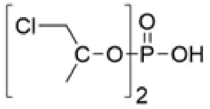	BCIPP	bis(1-chloro-2-propyl) hydrogen phosphate | BCIPP	bis(1-chloro-2-propyl) phosphate | BCIPP	bis(2-chloro-isopropyl) phosphate | BCIPP	bis (1-chloro-2-propyl) hydrogen phosphate | BCIPP
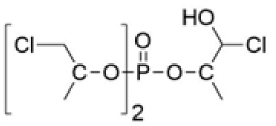	TCIPP-M3		TCIPP-M3	TCIPP-M3	1-chloro-3-hydroxypropan-2-yl bis (1-chloropropan-2-yl) phosphate | TCIPP-M3
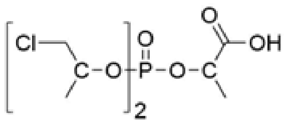	TCIPP-M2	bis(1-chloro-2-propyl) carboxy 2-ethyl phosphate | TCIPP-M2	TCIPP-M1	TCIPP-M1	carboxyethyl bis (1-chloro-2-propyl) phosphate | TCIPP-M1
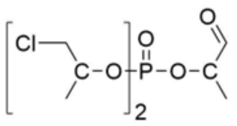					bis (1-chloropropan-2-yl) (-oxopropan-2-yl) phos phate | TCIPP-M2
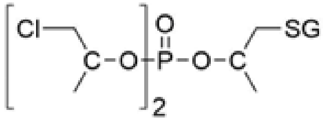		TCIPP-Glutathione			

As such, a unified naming and abbreviation convention for all the metabolites identified in previous as well as the current paper were proposed; considering previous authors preferences and reflecting the limitations of MS techniques (Text S1-Table 4).

Text S1-Table 4 Proposed abbreviations for TCPP metabolites used in this paper.

Structure	Name	Abbreviation
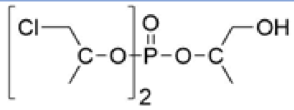	**B**is(2-**c**hloro**i**so**p**ropyl) **h**ydroxy-**i**so**p**ropyl **p**hosphate	BCIPHIPP
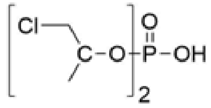	**B**is(2-**c**hloro**i**so**p**ropyl) phosphate	BCIPP
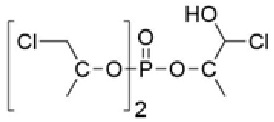	**B**is(2-**c**hloro**i**so**p**ropyl) **c**hloro-**h**ydroxy-**i**so**p**ropyl **p**hosphate	BCIPCHIPP
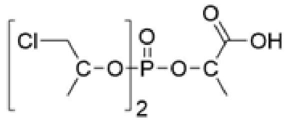	**B**is(2-**c**hloro**i**so**p**ropyl) **c**arboxy-**i**sopropyl **p**hosphate	BCIPCIPP
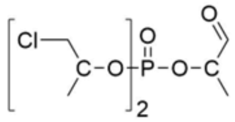	**B**is(2-**c**hloro**i**so**p**ropyl) **o**xo-**i**sopropyl **p**hosphate	BCIPOIPP
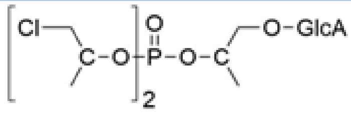	**B**is(2-**c**hloro**i**so**p**ropyl) **h**ydroxy-**i**so**p**ropyl **p**hosphate - glucuronic acid conjugate	BCIPHIPP-GlcA
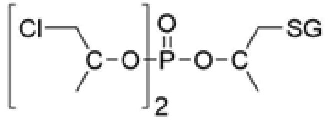	**B**is(2-**c**hloro**i**so**p**ropyl) **i**so**p**ropyl **p**hosphate – glutathione conjugate	BCIPIPP-SG

Text S2 Chemicals and reagents

Standards of BCIPP and BCIPHIPP and the internal standards BDCIPP-d10 and TCEP-d12 were custom synthesized by Dr. Vladimir Belov (Max Planck Institute, Gottingen, Germany). Methanol (LC-grade, ≥99.9) was obtained from Biosolve (Valkenswaard, the Netherlands). Formic acid (LC-MS grade, >98%), potassium phosphate, potassium hydroxide and β-glucuronidase (lyophilized powder from Escherichia coli, >10 000 000 unit/g) were purchased from Merck KGaA (Darmstadt, Germany). Ammonium acetate (LC-MS grade, ≥99%) was obtained from VWR (Leuven, Belgium). Ultrapure water was obtained from a PURELAB Flex system (ρ=18.2 MΩ/cm, Elga Veolia, Tienen, Belgium).

Text S3 Description of foam production process and activities of foam line workers and conversion workers

As described in de Kort. (2023), foam line workers perform a variety of activities in the foam production hall where the flexible foam production line is located (see Text S3-Figure 1 for a schematic diagram of such equipment). In this equipment polyol, diisocyanates, catalysts, stabilizers, additives, and flame retardants flame retardants such as TCPP (during production of combustion modified flexible foam), are combined in the mixing head before being poured onto a conveyer belt. On this conveyer the mixture simultaneously undergoes a blowing and gelling reaction, which causes the formation of gas and the polymerization of the mixture to form a solid material, respectively. This expanding and solidifying mixture travels on and between paper in a tunnel to expand to a block with a cross section of around 2x1 meter. The paper is removed towards the end of the line and the material is cut into long blocks (up to 120 m) or short blocks (2 m).

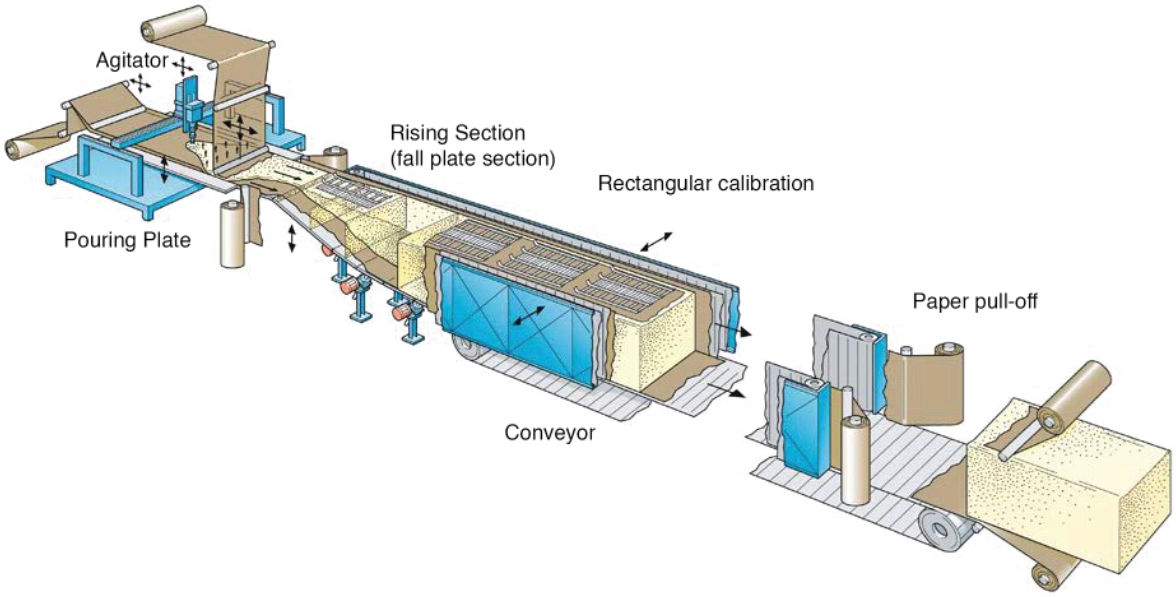

Text S3-Figure 1 Overview of the foam production process including the agitator, pouring plate, rising section, conveyor and paper pull-off in a system for continuous production of rectangular flexible foam blocks. 3-D representation of a system – without metering device and cut-off saw – for continuous production of flexible rectangular foam blocks by means of the QFM process (Image courtesy of the Hennecke Group)

The chemical reaction is exothermic and thus the material reaches temperatures of 120 – 150 °C depending on the starting temperature of the raw material and the formulation. Furthermore, contrary to rigid foam, flexible foam has an open cell structure increasing the emission potential of substances (including TCPP) from the material. The entire tunnel is encapsulated and fitted with powerful extraction ventilation to control the airborne concentration of the diisocyanates, such as toluene diisocyanate, which are potent respiratory sensitizers. During activities and in locations where the concentration of toluene diisocyanate as measured with direct reading instruments is too high, respiratory protective equipment (RPE) is worn. This will also reduce the exposure to TCPP.

Activities of foam line operators include monitoring the flow of material and making slight adjustments to the formulation from the mixing platform (Text S3-Figure 2A), visually inspecting the flowing material from the side of the tunnel and making manual manipulations when required (Text S3-Figure 2B), changing side paper rolls (Text S3-Figure 2C), and operating the cut-off saw (Text S3-Figure 2D). All activities involving potential contact with the uncured foam are performed with chemically resistant gloves.

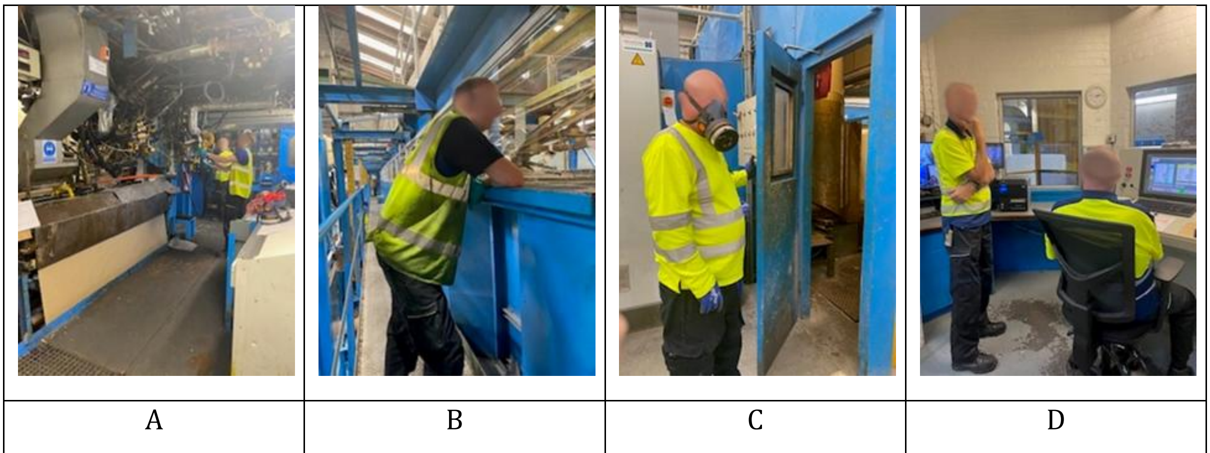

A	B	C	D

Text S3-Figure 2 Operations taking place in the foam hall by foam line operators in factory C007. A: worker on mixing platform observing the flow of material. B: worker monitoring the flow of foam in the tunnel from the side of the tunnel through glass windows. Ready to equip RPE, open the window, and perform manual manipulations (e.g. pulling up paper in case of side paper drift). C: foam line operator about to enter enclosure where side paper is removed to change the roll collecting the side paper. D: operators in the control room managing the saw towards the end of the line and subsequent automatic transport to the curing storage area.

Foam coming from the foam line is transported to a curing area where it is left to cool down and finalize its polymerization reaction. Once cured, the foam can be sold as such or further cut to specific shapes in specific equipment. Such operations are called conversion operations and are performed by workers in the conversion departments. Since the foam is at ambient temperature the emission of TCPP from the material will be lower. However, there is more extensive dermal contact between conversion workers and the foam. Gloves may be worn here for mechanical hazards (e.g. cutting hazards) which would potentially reduce exposure, but these gloves are not chemically resistant gloves. Furthermore, the level of ventilation is not much beyond general hall ventilation and no respiratory protective equipment is worn.

Text S4 Quantitative analysis of PFR metabolites in urine

Concentrations of BCIPP and BCIPHIPP were analyzed following a previously validated method (Bastiaensen et al., 2018). Briefly, 1 mL of urine sample was spiked with 5 ng of mass-labeled internal standard mixture (TCEP-d12 for BCIPHIPP quantification, BDCIPP-d10 for BCIPP quantification, Table S2), adjusted with phosphate buffer to pH 6 and deconjugated with β-glucuronidase (2 mg/mL) during a 2 hour incubation at 37 °C. Sample extraction was done on Bond-Elut C18 cartridges (3 mL, 200 mg, Agilent, Santa Clara, USA), conditioned with 3 mL of methanol followed by 2 mL of MilliQ water. Analytes were eluted with 3 mL of methanol, evaporated until near dryness and reconstituted in 100 µL MeOH:MilliQ 1:1. Extracts were then filtered using a micro-centrifuge filter (0.2 µm nylon, VWR, Leuven, Belgium) and injected on a Agilent 1290 Infinity liquid chromatography system coupled to a triple quadrupole mass spectrometer (ESI-6495). Separation was achieved using a Biphenyl column (2.1 mm x 100 mm, 2.6 µm; Phenomenex Kinetex, Torrance, USA) with 5 mM ammonium acetate in H2O with 2 % methanol and 5 mM ammonium acetate in MeOH with 2 % H2O as mobile phase A and mobile phase B, respectively. The instrument was operated in dynamic multiple reaction monitoring in positive and negative ionization mode. Details of the analytical method are reported in Bastiaensen et al. (2018) with adapted parameters for analysis on the ESI-6495 shown in Table S2 and S3.

Text S5 Toxicity of TCPP

Current health based guidance values (HBGV) have used the endpoint of increased liver weight/hepatocyte hypertrophy as point of departure. The EU RAR 2008, Van den Eede et al. (2011) and the ECHA 2018 screening report based their DNEL on a 90-day study in rats from Stauffer Chemical Company (1981). Rats were dosed with TCPP through food at doses of 0, 52, 160, 481, and 1349 mg/kg/day for males and 0, 62, 171, 570, and 1745 mg/kg/day for females. No treatment related mortalities were reported, and no adverse clinical effects such as weight gain and hematology parameters were observed. The mean absolute and relative liver weights were significantly increased in males in all dose groups and in females in the two highest dose groups. In the highest dose groups in both males and females, periportal hepatocyte swelling was found. Relative kidney weights were significantly higher in the two highest dose groups in male rats. In addition, mild degenerative histopathological changes in the kidneys were seen in these dose groups. However, these changes were regarded as male rat specific nephropathies and thus not considered relevant for humans. In all dose groups in males, mild thyroid follicular cell hyperplasia was observed, while in females this was only seen in the highest dose group. The increase in absolute and relative liver weight and the observation of mild thyroid follicular cell hyperplasia, was considered by the EU RAR (2008) and ECHA Screening Report to establish a Lowest Observed Adverse Effect Level (LOAEL) of 52 mg/kg bw/day; while Van den Eede et al. (2011) considered this to be a NOAEL. Furthermore, Van den Eede et al. (2011) used an assessment factor (AS) of 10 000, which some more conservative authors used at the time to cover additional (perceived) uncertainty. For example, this approach was also used for the creation of special “screening values” (United States Environmental Protection Agency, 2012).

The EU RAR (2008) implied the need for an assessment factor of 10 to account for interspecies differences, and a factor of 5 and 10 for intraspecies differences for workers and the general population, respectively; which is in line with regulatory guidance. The EU RAR states that normally a factor 3 would be needed to extrapolate from the LOAEL to a NOAEL, but that is not necessary here since “the LOAEL derived from the repeat dose toxicity study was based on liver weight changes which are not considered to be particularly toxicologically significant and the LOAEL is probably quite close to the NOAEL”. The usual assessment factor of 2 for the extrapolation from a 90-day study to chronic duration was also not applied as it was “not considered necessary, as relatively similar effects on the liver, at doses of a comparable order of magnitude were observed in both the 28-day and the 90-day studies and so it is felt that exposure duration is not significant”. This approach resulted in a total AS of 50 for workers and 100 for the general population. The ECHA Screening Report, 2018 used the same AS for inter- and intra-species differences as the EU RAR, but adds on to this a factor of 6 for the dose response relationship and applies a factor 2 to extrapolate from subchronic to chronic exposure duration resulting in a total AS of 600 for workers and 1200 for the general population.

The US EPA (United States Environmental Protection Agency, 2012) derived its HBGV for TCPP from the subchronic dose-range finding part of the NTP study to TCPP in mice (NTP, 2023). Mice were dosed with TCPP through food for 14 weeks with 0, 219, 456, 737, 2470, and 4410 mg/kg day for males and 0, 198, 420, 906, 1930, and 3590 mg/kg day for females. No treatment related mortalities or abnormal clinical signs were reported. Body weights were decreased in both male and female mice in a time- and dose-dependent manner. Relative liver weight was statistically significant increased at all doses in males and at 198, 906, 1930 and 3950 mg/kg bw/day in female mice. Hepatocyte hypertrophy was observed in male mice at doses higher than 456 mg/kg bw/day and in female mice at doses higher than 906 mg/kg bw/day. US EPA performed benchmark dose modelling to derive a BMDL10 of 138 mg/kg bw/day as point of departure for further risk assessment. The US EPA report used 10 as an assessment factor for inter- and intra-species differences (100 in total) and added a factor of 10 for database uncertainties; a factor used for substances with limited study information available. In

addition, they added another factor 10 to correct for the fact that they have used a subchronic study. The US EPA also explicitly mentioned that it does not use a dose-response factor since it uses a BMDL10 which addresses this issue.

All the above limit values were based on increased liver weight and the supposition that this might be a prelude to carcinogenicity, should the exposure duration be extended to the whole lifetime. The United States National Toxicology Program (US NTP) has recently performed a chronic (2 year) study in rats and mice to evaluate the carcinogenic potential of TCPP and to derivate toxicological benchmarks. These findings will be evaluated in Text S5-2 in line with ECHA Guidance R12 (European Chemicals Agency, 2015) to derive DNELs, a standardized regulatory accepted form of HBGVs. Some of the authority reports, namely EU RAR and the ECHA Screening Report, mention a concern for reproductive and/or developmental toxicity, for which newer studies discussed in Text S5-1 have become available.

Text S5-1 Reprotoxicity and developmental toxicity

A prenatal developmental toxicity study to TCPP was conducted in rabbits, in which no adverse maternal and developmental effects were observed even in the highest dose group of 500 mg/kg bw/day (European Chemicals Agency, 2017). In the NTP study on reprotoxicity of TCPP (NTP, 2020), female rats were administered 0, 162.5, 325 or 650 mg/kg bw/day TCPP by oral gavage from gestational day 6 to gestational day 20. No significant effects were observed on post implantation loss, mean fetal body weights or fetal sex ratio. In addition, no biologically relevant exposure-related malformations were observed in external, visceral and skeletal fetal exams of TCPP-exposed fetuses. This led to the conclusion that there is no evidence of reprotoxicity and developmental toxicity of TCPP in rats (NTP, 2020). As such, no DNELs were derived for this endpoint.

Text S5-2 Carcinogenicity

The NTP conducted a 2-year toxicity study with rats and mice to study potential carcinogenic toxicity of TCPP (NTP, 2023). Rats were chronically exposed to TCPP through feed with doses of 0, 2500, 5000, 10000, and 20000 ppm which resulted in doses of 0, 141, 294, 626 or 1155 mg/kg bw/day for male rats and 0, 156, 323, 674 or 1295 mg/kg bw/day for female rats. Male mice were chronically exposed to 0, 160, 330 or 711 mg/kg bw/day in food, while female mice were exposed to 0, 329, 673 or 1491 mg/kg bw/day in food.

In rats, several statistically significant non-neoplastic effects were observed in the liver of both sexes. Bile duct hyperplasia was an apparent benign adaptation to the increased flux of material through enterohepatic circulation (see Text S6). Pigmentation was observed at the highest dose group in both sexes and similarly a result of the saturation of the entero-hepatic circulation. There were several foci which reached statistical significance in the two highest dose groups (basophilic in male and eosinophilic in females) or only the highest dose group (eosinophilic and mixed-cell in males). These foci are pre-neoplastic according to the US NTP Histopathological atlas which are “relatively common in chronic studies but uncommon in 90-day studies” (Maronpor, n.d.), which implies that these should be of greater concern when observed in subchronic studies and less concern in chronic studies. An overview of the incidence of these non-neoplastic findings in historical control groups was compiled and can be found in Text S5-2-Table 1, Text S5-2-Table 2 and Text S5-2-Figure 1. In the groups dosed with 2500 and 5000 ppm TCPP in feed, no adverse non-neoplastic effects were observed.

Text S5-2-Table 1 Frequency of occurrence of relevant non-neoplastic findings in male SD rat control groups of previous NTP studies. Data were collected from the P03 table of previous NTP studies conducted in male SD rats.

P03 Data Table	Basophilic focus (%)	Clear Cell focus (%)	Eosinophilic focus (%)	Mixed Cell focus (%)	Pigment (%)	Bile Duct Hyperplasia (%)	Bile Duct Cyst 9%)
TR-602	10 (5/50)	22 (11/50)	10 (5/50)	4 (2/50)	0 (0/50)	14 (7/50)	2 (1/50)
TR-601	12 (6/50)	58 (29/50)	2 (1/50)	2 (1/50)	0 (0/50)	18 (9/50)	4 (2/50)
TR-601	2 (1/50)	48 (24/50)	8 (4/50)	0 (0/50)	0 (0/50)	26 (13/50)	0 (0/50)
TR-600	8 (4/49)	49 (24/49)	0 (0/49)	10 (5/49)	0 (0/49)	31 (15/49)	4 (2/49)
TR-599	4 (2/50)	28 (14/50)	6 (3/50)	6 (3/50)	0 (0/50)	84 (42/50)	2 (1/50)
C20614B	2 (1/50)	70 (35/50)	6 (3/50)	0 (0/50)	0 (0/50)	48 (24/50)	0 (0/50)
TR-597	2 (1/50)	58 (29/50)	12 (6/50)	0 (0/50)	0 (0/50)	40 (20/50)	0 (0/50)
TR-595	1 (1/90)	1 (1/90)	9 (8/90)	36 (32/90)	0 (0/90)	46 (41/90)	3 (3/90)
TR-594	4 (2/50)	80 (40/50)	2 (1/50)	4 (2/50)	0 (0/50)	64 (32/50)	0 (0/50)
TR-592	0 (0/50)	36 (18/50)	8 (4/50)	0 (0/50)	0 (0/50)	0 (0/50)	0 (0/50)
TR-584	6 (3/50)	28 (14/50)	20 (10/50)	8 (4/50)	0 (0/50)	14 (7/50)	0 (0/50)

The table contains hyperlinks to the original P03 Tables as reported for TCPP (TR-602), Di(2-ethylhexyl) phthalate (TR-601), dibutyl phthalate (TR-600), sodium tungstate dihydrate (TR-599), perfluorooctanoic acid (C20614B), 2-hydroxy-4-methoxybenzophenone (TR-597), cell phone radiation (CDMA) (TR-595), p-chloro-a,a,a-trifluorotoluene (TR-594), zinc carbonate (TR-592), indole-3-carbinol (TR-584).

Text S5-2-Table 2 Frequency of occurrence of relevant non-neoplastic findings in female SD rat control groups of previous NTP studies. Data were collected from the P03 table of previous NTP studies conducted in female SD rats.

P03 Data Table	Basophilic focus (%)	Clear Cell focus (%)	Eosinophilic focus (%)	Mixed Cell focus (%)	Pigment (%)	Bile Duct Hyperplasia (%)	Bile Duct Cyst (%)
TR-602	10 (5/50)	22 (11/50)	10 (5/50)	4 (2/50)	0 (0/50)	14 (7/50)	2 (1/50)
TR-601	12 (6/50)	16 (8/50)	14 (7/50)	6 (3/50)	6 (3/50)	12 (6/50)	8 (4/50)
TR-601	8 (4/49)	29 (14/49)	6 (3/49)	0 (0/49)	0 (0/49)	18 (9/49)	0 (0/49)
TR-600	8 (4/50)	22 (11/50)	4 (2/50)	6 (3/50)	0 (0/50)	10 (5/50)	10 (5/50)
TR-599	0 (0/50)	16 (8/50)	4 (2/50)	4 (2/50)	0 (0/50)	2 (1/50)	4 (2/50)
C20614	22 (11/50)	22 (11/50)	16 (8/50)	2 (1/50)	6 (3/50)	32 (16/50)	18 (9/50)
TR-597	12 (6/50)	24 (12/50)	30 (15/50)	2 (1/50)	0 (0/50)	0 (0/50)	4 (2/50)
TR-595	12 (11/90)	2 (2/90)	10 (9/90)	32 (29/90)	0 (0/90)	10 (9/90)	12 (11/90)
TR-594	14 (7/50)	32 (16/50)	14 (7/50)	12 (6/50)	2 (1/50)	28 (14/50)	8 (4/50)
TR-592	2 (1/50)	4 (2/50)	0 (0/50)	0 (0/50)	0 (0/50)	0 (0/50)	0 (0/50)
TR-584	28 (14/50)	12 (6/50)	0 (0/50)	10 (5/50)	2 (1/50)	4 (2/50)	4 (2/50)

The table contains hyperlinks to the original P03 Tables as reported for TCPP (TR-602), Di(2-ethylhexyl) phthalate (TR-601), dibutyl phthalate (TR-600), sodium tungstate dihydrate (TR-599), perfluorooctanoic acid (C20614B), 2-hydroxy-4-methoxybenzophenone (TR-597), cell phone radiation (CDMA) (TR-595), p-chloro-a,a,a-trifluorotoluene (TR-594), zinc carbonate (TR-592), indole-3-carbinol (TR-584).

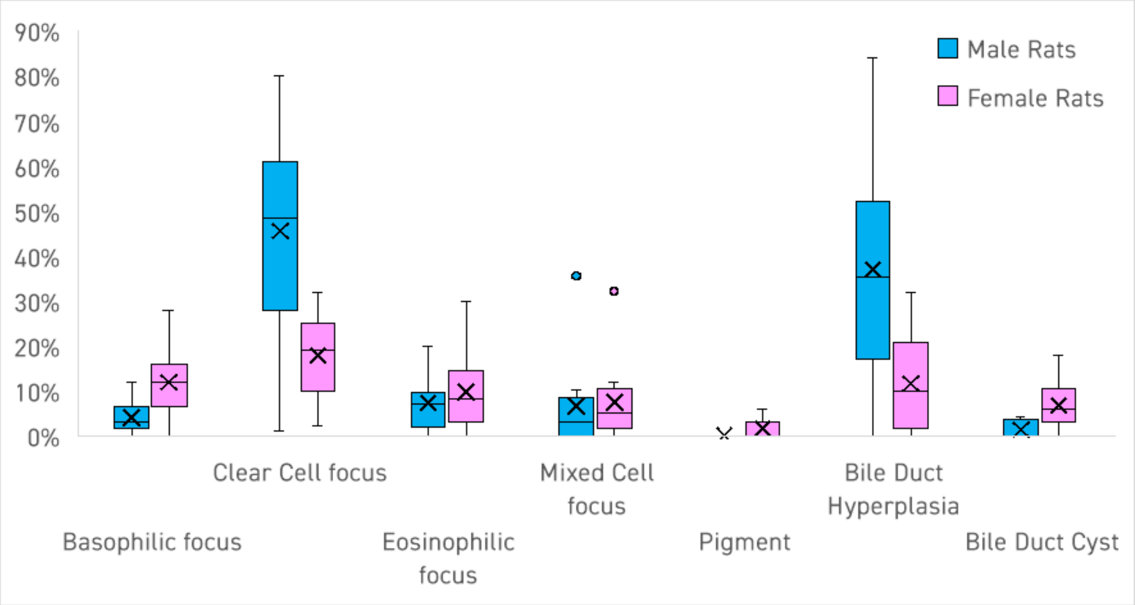

Text S5-2-Figure 1 Box plots of relevant non-neoplastic findings in control groups in previous NTP studies in male and female SD rats. Data were obtained from the P03 tables for Di(2-ethylhexyl) phthalate (TR-601), dibutyl phthalate (TR-600), sodium tungstate dihydrate (TR-599), 2-hydroxy-4-methoxybenzophenone (TR-597), cell phone radiation (CDMA) (TR-595), p-chloro-a,a,a-trifluorotoluene (TR-594), zinc carbonate (TR-592), indole-3-carbinol (TR-584).

In male rats, a positive trend for incidence of hepatocellular adenoma or carcinoma (combined) was seen. However, this finding was not statistically significant in pairwise comparison and therefore it was labeled only as 'some evidence' of carcinogenicity. In the 2500 and 5000 ppm dose groups there does not seem to be any alteration in the occurrence of any non-neoplastic and neoplastic lesions, indicating that if there is any effect on the liver male SD rats it is clearly a threshold-based phenomenon; as such the dose of 5000 ppm in feed (294 mg/kg/d) is taken forward as a NOAEL in male rats. In female rats, a non-significant increase in the incidence of hepatocellular adenomas was observed, which the NTP labeled as an 'equivocal finding'. This equivocal finding is based on 1/50, 3/50, 0/50, 3/50, and 3/50 animals developing hepatocellular adenoma in the 0, 2500, 5000, 10000, and 20000 ppm dose groups, as such it should be treated as more of a proof of an absence of neoplastic effect in the liver of the female rat. A positive trend for incidence of uterine adenoma or adenocarcinoma was identified based on histopathology, but this trend was also not significant in pairwise comparison. These findings led to 'some evidence' of carcinogenicity in female rats.

Recent reviews of data for other substances however reveal that depending on mode of action, uterine tumors in rats may not be significant for humans (Klaunig et al., 2016; Wikoff et al., 2016). Because there was no dose-response that allows for a clear identification of a NOAEL in female rats, it was decided to perform benchmark dose modeling. A benchmark dose modelling was performed using The National Institute for Public Health and the Environment of the Netherlands (RIVM) software for dose-response modelling and benchmark dose analysis (PROAST) to derive a benchmark dose of 5% (BMD5) of 259 mg/kg/day, which is considered an equivalent PoD to a NOAEL according to ECHA Guidance (European Chemicals Agency, 2012; National Institute for Public Health and the Environment, 2024).

In male mice, no statistically significant non-neoplastic effects were observed in the liver in any of the dosed groups. A significant increase in the incidence of hepatocellular adenoma was observed compared to controls (10%, 28%, 34%, and 28% in the 0, 156, 323, 674 or 1295 mg/kg bw/day dosed groups), however this increase was seen across all dose groups without a clear dose-dependent increase in incidence. This observation could be because the incidences of hepatocellular carcinoma

in the used control groups were unusually low compared to historical control groups from previous NTP studies reporting hepatocellular carcinoma in male rats. When NTP compared the incidence with a dataset of historical controls, the incidence of hepatocellular carcinoma in the different dose groups was similar to the incidence in the historical controls. What the report fails to mention is that in the dataset used for comparison, they have included only three studies as comparison with one of them being on TCPP. Therefore, the conclusion made in the NTP report that the incidence in the control group is merely "at the lower end of the NTP's historical range for this neoplasm", should be treated with caution. A broader dataset of historical controls was compiled in Text S5-2-Table 3, Text S5-2-Table 4, Text S5-2-Figure 2 and Text S5-2-Figure 3 showing incidences of neoplasms in B6C3F1/N mice used by NTP. In the historical control groups, the average incidence of hepatocellular carcinoma in male mice is 26%, meaning the observed incidence in the dosed groups is well within the historical control ranges, and the incidence of 10% in the control group is well below the historical control range. Therefore, while the NTP report concludes that there is 'some evidence' of carcinogenic activity in male mice, we do not consider there is enough evidence of carcinogenic effects in male mice to bring forward a NOAEL to derive a DNEL for male mice.

Text S5-2-Table 3 Occurrence of Basophilic, Clear Cell, Eosinophilic, and Mixed Cell Focus in Control Groups of Male and Female B6C3F1/N mice of previous NTP studies. Data were collected from the P03 table of previous NTP studies conducted in male and female B6C3F1/N mice.

P03 Table	Male Mice				Female Mice			
	Basophilic (%)	Clear Cell (%)	Eosinophilic (%)	Mixed Cell (%)	Basophilic (%)	Clear Cell (%)	Eosinophilic (%)	Mixed Cell (%)
TR-602	8 (4/50)	26 (13/50)	26 (13/50)	0 (0/50)	4 (2/50)	6 (3/50)	2 (1/50)	0 (0/50)
TR-603					12 (6/50)	6 (3/50)	14 (7/50)	0 (0/50)
TR-594	8 (4/50)	30 (15/50)	22 (11/50)	30 (15/50)	2 (1/50)	6 (3/50)	8 (4/50)	20 (10/50)
TR-600	8 (4/50)	20 (10/50)	14 (7/50)	20 (10/50)	12 (6/50)	0 (0/50)	2 (1/50)	4 (2/50)
TR-597	10 (5/50)	24 (12/50)	14 (7/50)	6 (3/50)	8 (4/50)	2 (1/50)	8 (4/50)	0 (0/50)
TR-599	4 (2/50)	32 (16/50)	46 (23/50)	16 (8/50)	4 (2/50)	2 (1/50)	16 (8/50)	2 (1/50)
TR-596	1 (1/90)	31 (28/90)	4 (4/90)	2 (2/90)	4 (4/90)	1 (1/90)	2 (2/90)	6 (5/90)
TR-584	4 (2/50)	14 (7/50)	58 (29/50)	2 (1/50)	8 (4/50)	6 (3/50)	32 (16/50)	4 (2/50)
TR-589	12 (6/50)	20 (10/50)	28 (14/50)	4 (2/50)	2 (1/50)	0 (0/50)	6 (3/50)	0 (0/50)
TR-587	18 (9/50)	22 (11/50)	40 (20/50)	14 (7/50)	16 (8/50)	6 (3/50)	22 (11/50)	8 (4/50)
TR-585	16 (8/50)	48 (24/50)	48 (24/50)	18 (9/50)	10 (5/50)	6 (3/50)	46 (23/50)	8 (4/50)
TR-590	8 (4/50)	28 (14/50)	8 (4/50)	2 (1/50)	0 (0/50)	12 (6/50)	4 (2/50)	2 (1/50)
TR-593	14 (7/50)	4 (2/50)	8 (4/50)	8 (4/50)	2 (1/50)	2 (1/50)	2 (1/50)	4 (2/50)
TR-586	2 (1/50)	20 (10/50)	28 (14/50)	12 (6/50)	12 (6/50)	2 (1/50)	12 (6/50)	4 (2/50)
TR-591	22 (11/50)	28 (14/50)	16 (8/50)	6 (3/50)	12 (6/50)	0 (0/50)	6 (3/50)	2 (1/50)

The table contains hyperlinks to the original P03 Tables as reported for TCPP (TR-602), Black Cohosh (TR-603), p-Chloro-a,a,a-trifluorotoluene (TR-594), Dibutyl Phthalate (TR-600), 2-Hydroxy-4-methoxybenzophenone (TR-597), Sodium Tungstate Dihydrate (TR-599), Radiofrequency Radiation (TR-596), Indole-3-carbinol (TR-584), PentaBDE (TR-589), TBBPA (TR-587), Green Tea Extract (TR-585), Antimony trioxide (TR-590), 2,3-Butanedione (TR-593), CIMSTAR 3800 (TR-586), and TRIM VX (TR-591).

Text S5-2-Table 4 Occurrence of hepatocellular adenoma, carcinoma, and adenoma and carcinoma combined in Control Groups of Male and Female B6C3F1/N mice of previous NTP studies. Data were collected from the P08 table of previous NTP studies conducted in male and female B6C3F1/N mice.

P08 Table	Male Mice			Female Mice		
	Adenoma (%)	Carcinoma (%)	Combined (%)	Adenoma (%)	Carcinoma (%)	Combined (%)
TR-602	42 (21/50)	10 (5/50)	46 (23/50)	22 (11/50)	2 (1/50)	24 (12/50)
TR-603				12 (6/50)	6 (3/50)	16 (8/50)
TR-594	50 (25/50)	16 (8/50)	60 (30/50)	24 (12/50)	14 (7/50)	36 (18/50)
TR-600	36 (18/50)	20 (10/50)	48 (24/50)	6 (3/50)	2 (1/50)	8 (4/50)
TR-597	43 (21/49)	16 (8/49)	53 (26/49)	6 (3/50)	2 (1/50)	8 (4/50)
TR-599	52 (26/50)	30 (15/50)	66 (33/50)	22 (11/50)	4 (2/50)	26 (13/50)
TR-596	58 (52/90)	31 (28/90)	74 (67/90)	21 (19/89)	9 (8/89)	28 (25/89)
TR-584	52 (26/50)	24 (12/50)	70 (35/50)	14 (7/50)	12 (6/50)	24 (12/50)
TR-589	46 (23/50)	36 (18/50)	62 (31/50)	10 (5/50)	8 (4/50)	16 (8/50)
TR-587	64 (32/50)	22 (11/50)	78 (39/50)	26 (13/50)	4 (2/50)	30 (15/50)
TR-585	70 (35/50)	30 (15/50)	80 (40/50)	24 (12/50)	10 (5/50)	28 (14/50)
TR-590	55 (33/60)	27 (16/60)	70 (42/60)	18 (11/60)	10 (6/60)	23 (14/60)
TR-593	34 (17/50)	34 (17/50)	62 (31/50)	12 (6/50)	10 (5/50)	22 (11/50)
TR-586	48 (24/50)	22 (11/50)	64 (32/50)	28 (14/50)	20 (10/50)	40 (20/50)
TR-591	46 (23/50)	42 (21/50)	68 (34/50)	18 (9/50)	14 (7/50)	30 (15/50)

The table contains hyperlinks to the original P08 Tables as reported for TCPP (TR-602), Black Cohosh (TR-603), p-Chloro-a,a,a-trifluorotoluene (TR-594), Dibutyl Phthalate (TR-600), 2-Hydroxy-4-methoxybenzophenone (TR-597), Sodium Tungstate Dihydrate (TR-599), Radiofrequency Radiation (TR-596), Indole-3-carbinol (TR-584), PentaBDE (TR-589), TBBPA (TR-587), Green Tea Extract (TR-585), Antimony trioxide (TR-590), 2,3-Butanedione (TR-593), CIMSTAR 3800 (TR-586), and TRIM VX (TR-591).

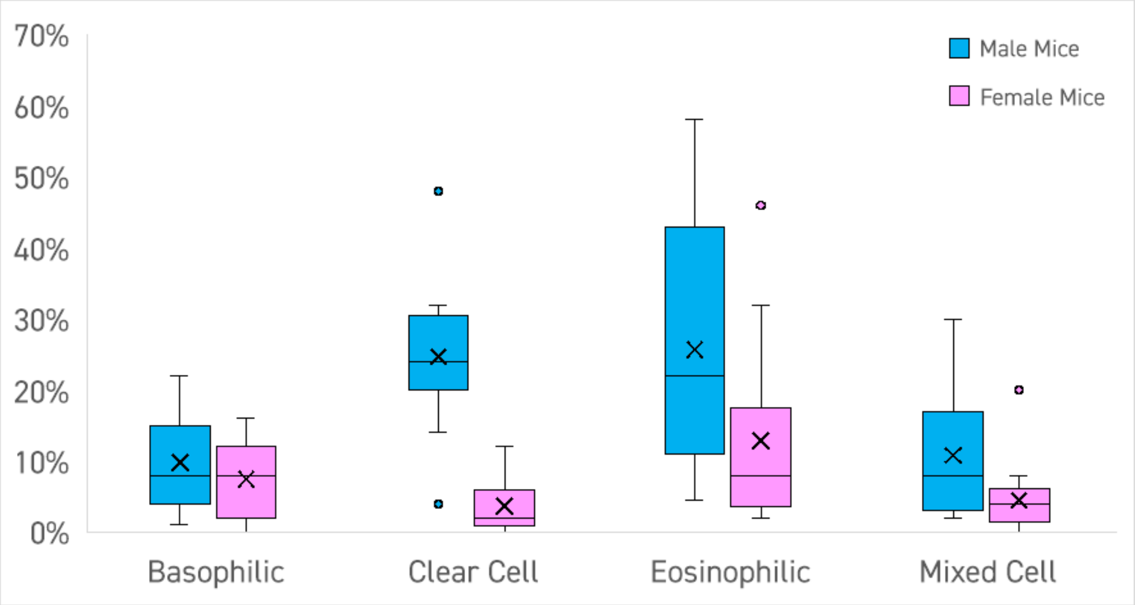

Text S5-2-Figure 2 Occurrence of Basophilic, Clear Cell, Eosinophilic, and Mixed Cell Focus in Control Groups in previous NTP studies in Male and Female B6C3F1/N mice. Data were obtained from the P03 tables for Black Cohosh (TR-603), p-Chloro-a,a,a-trifluorotoluene (TR-594), Dibutyl Phthalate (TR-600), 2-Hydroxy-4-methoxybenzophenone (TR-597), Sodium Tungstate Dihydrate (TR-599), Radiofrequency Radiation (TR-596), Indole-3-carbinol (TR-584), PentaBDE (TR-589), TBBPA (TR-587), Green Tea Extract (TR-585), Antimony trioxide (TR-590), 2,3-Butanedione (TR-593), CIMSTAR 3800 (TR-586), and TRIM VX (TR-591).

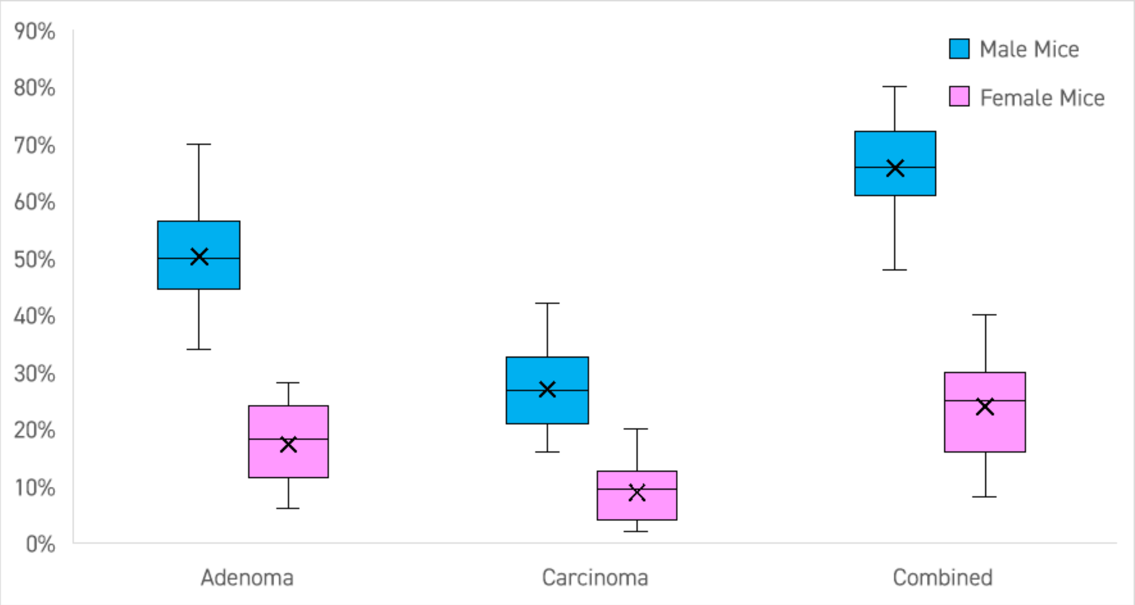

Text S5-2-Figure 3 Occurrence of hepatocellular adenoma, carcinoma, and adenoma and carcinoma combined in Control Groups in previous NTP studies in Male and Female B6C3F1/N mice. Data were obtained from the P08 tables from Black Cohosh (TR-603), p-Chloro-a,a,a-trifluorotoluene (TR-594), Dibutyl Phthalate (TR-600), 2-Hydroxy-4-methoxybenzophenone (TR-597), Sodium Tungstate Dihydrate (TR-599), Radiofrequency Radiation (TR-596), Indole-3-carbinol (TR-584), PentaBDE (TR-589), TBBPA (TR-587), Green Tea Extract (TR-585), Antimony trioxide (TR-590), 2,3-Butanedione (TR-593), CIMSTAR 3800 (TR-586), and TRIM VX (TR-591).

Finally, in female mice, non-neoplastic lesions such as eosinophilic focus seems to be dose dependently statistically significantly increasing. However, here it should be noted that the frequency of eosinophilic focus in the control group (2%) is very low compared to an extended set of historical controls (13%; see Text S5-2 Table 3 and Text S5-2 Figure 2). The frequency in the lowest dosed group of 2500 ppm (14%) is in fact close to these historical control values and even the higher dosed groups (26 and 32%) are within the historical control ranges, albeit less convincingly.

Furthermore, hepatocellular cytoplasmic alterations seem to occur at the highest dose group (10 000 ppm). The NTP report indicates that the observed cytoplasmic alteration is indicative of lower glycogen levels in the liver cells of the female B6C3F1/N mice, indicative of disruption of normal function of the liver. Here, it should be recalled that the rats with a comparable dose (in mg/kg bw/d) showed pigmentation within the liver also indicative for adaptation.

With regards to the neoplastic effect, significant increases were observed in the incidence of hepatocellular adenoma, hepatocellular carcinoma and hepatocellular adenoma or carcinoma (combined) with a dose-dependent response. This result led NTP to conclude ‘clear evidence’ for carcinogenic activity in female mice.

It is important to note that the larger body of historical controls, compiled in Text S5-2-Table 4 and Text S5-2-Figure 3, the average incidence rate in controls for adenoma, carcinoma, and combined adenoma and carcinoma is 17%, 9%, and 24%, respectively. Taking this into account, it remains clear that the statistically significant increase in incidence of these tumors (46, 20, and 58%, respectively) compared to the study controls in the highest dose group (10 000 ppm; 1491 mg/kg/d) is elevated. The incidence (26, 10, and 32%, respectively) in the second highest dose group (5000 ppm; 673

mg/kg/d) is not statistically significantly elevated compared to study controls and while slightly higher than historical controls is within the historical control ranges. The incidence of neoplasms (10, 4, and 14%, respectively) in the lowest dose group (2500 ppm; 329 mg/kg/d) was lower than the historical controls. Taken together the NOAEL could be set at 673 mg/kg/d for neoplastic effect, however a NOAEL of 329 mg/kg bw/day would be more conservative and cover potential non-neoplastic effects in the liver of female mice.

Text S6 Toxicokinetic behavior of TCPP

The first investigation into the toxicokinetics of TCPP was performed in 1984 by the Stauffer Chemical Company, public summary available in the ECHA registration dossier (European Chemicals Agency, 1984). Groups of 5 – 6 Sprague-Dawley (SD) rats were given a single dose of 14C-TCPP. The groups consisted of and received: male 20 mg/kg intravenously (M20IV), male 20 mg/kg orally (M20PO), male 200 mg/kg orally (M200PO), and female 200 mg/kg orally (F200PO). Plasma was collected after 5, 15, 30 minutes as well as at 1, 2, 4, 6 ,8, 12, 24, 36, 48, 72, 96, 120, and 144 hours and TCPP levels were measured with liquid scintillation counting revealing a biphasic excretion pattern (see Text S6-Figure 1). Based on a comparison of the area under the curve of the measured blood plasma levels of animals dosed with 20 mg/kg TCPP by the intravenous route and the oral route the authors concluded that “absorption” of TCPP was 27.6 ± 5.4%. However, it should be noted that in fact this is the “systemic bioavailability” following oral dosing. Absorption may be higher but first pass metabolism and/or excretion of TCPP (or its metabolites) by the liver into bile (see below), may result in lower systemic bioavailability.

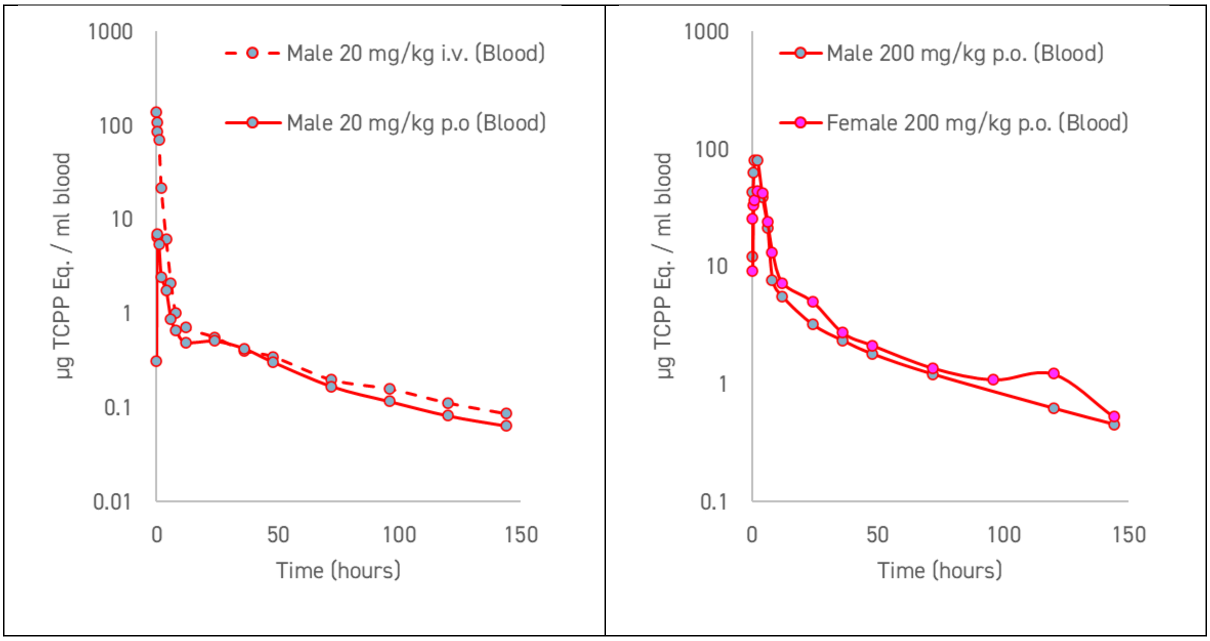

Text S6-Figure 1 Blood plasma concentration of TCPP and/or its metabolites as measured with liquid scintillation counting following administration of 20 mg/kg radiolabelled TCPP by intravenous injection or per oral (left) in male rats and administration of 200 mg/kg radiolabelled TCPP per oral route in male and female rats (right). Source: Stauffer Chemical Company (1984).

Urine, feces, and expired air were collected at 6, 12, 24, 36, 48, 72, 96, 120, 144, 168, and 192 hours after dosing and measured with scintillation counting (see Text S6-Figure 2 and Text S6-Figure 3). In the 20 mg/kg dose groups, radioactivity was detected in both urine and feces, however urinary excretion in orally dosed animals (49%) was lower than in those dosed intravenously (63%). This could be because less dose is absorbed or because the dose absorbed and delivered to the liver via the portal vein and thus more substantially extracted into bile (or due to a combination of effects). Increasing the oral dose to 200 mg/kg resulted in a greater fraction of the dose being excreted urinarily at the expense of fecal excretion. This could be the result of more extensive metabolism resulting in more hydrophilic compounds with greater affinity of the blood which would then be directed to urine.

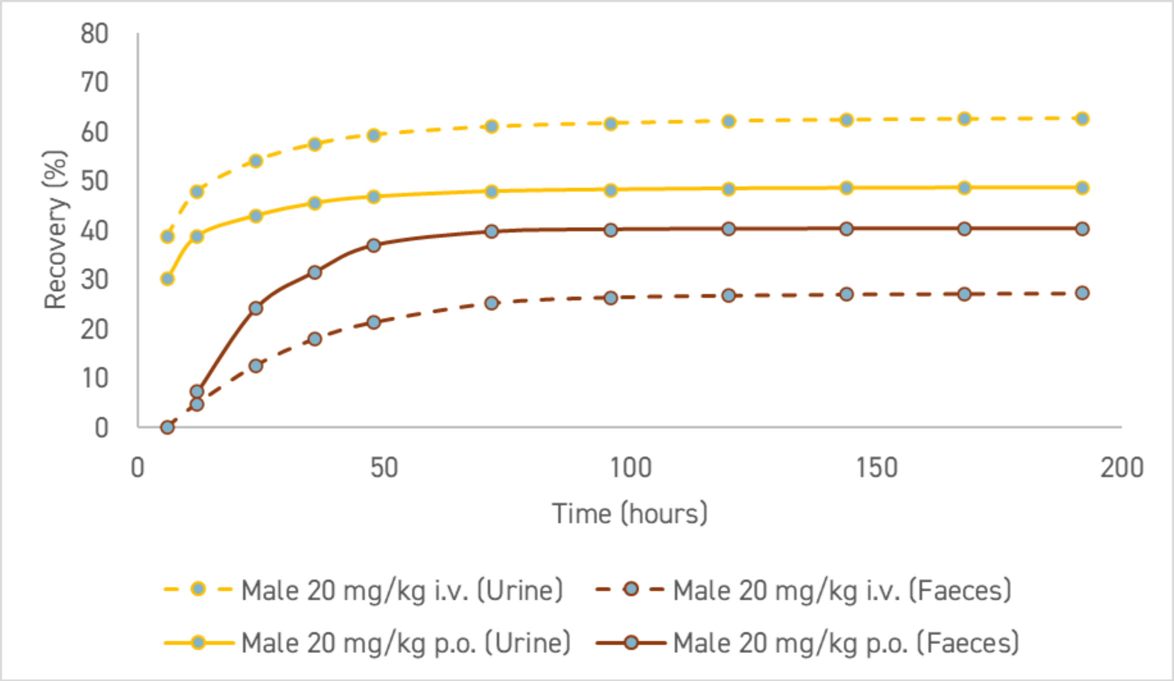

Text S6-Figure 2 Recovery of radiolabeled TCPP in urine and faeces over time in male SD rats dosed with 20 mg/kg radiolabeled TCPP by either intravenous or oral route. Data were obtained from the Stauffer Chemical Company 1984.

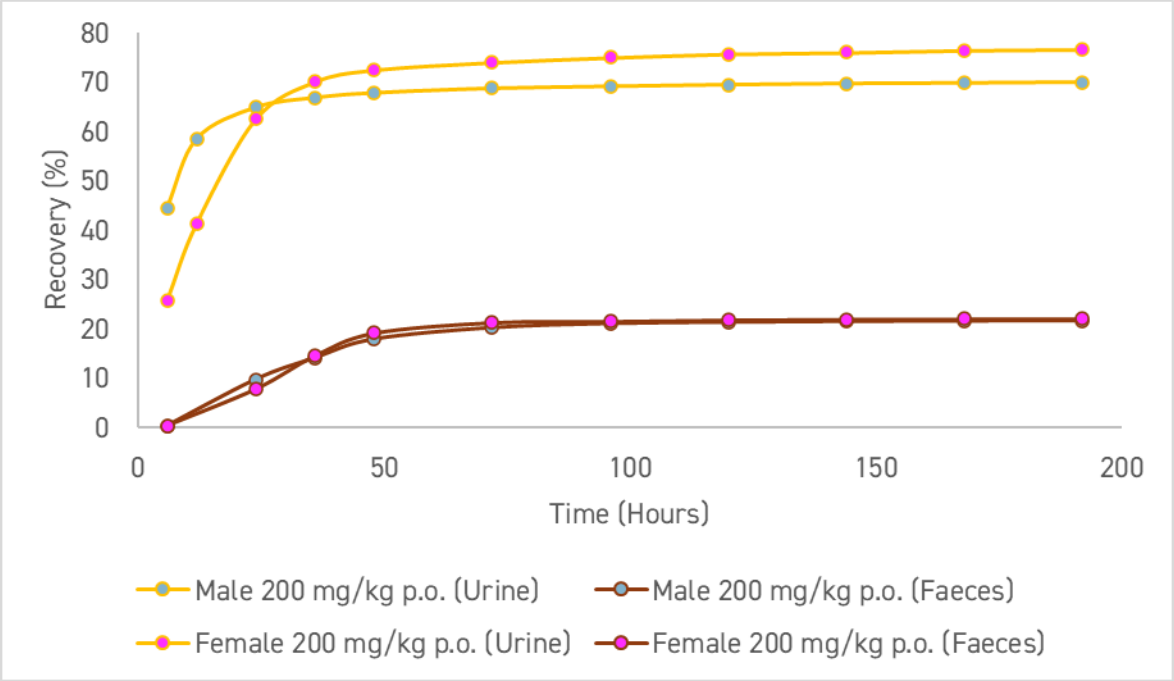

Text S6- Figure 3 Recovery of radiolabeled TCPP in urine and faeces over time in male and female SD rats dosed with 200 mg/kg radiolabeled TCPP by oral route. Data were obtained from the Stauffer Chemical Company, 1984.

Composite samples of urine or feces and urine were analyzed with a combination of thin layer chromatography, GC-MS, and GC-FID/RAD, to determine that the excreted dose consisted of 50% Bis(2-chloroisopropyl) carboxy-isopropyl phosphate (BCIPCIPP), 12% BCIPP, and unchanged <2% TCPP. The authors postulated that TCIPP would undergo α-heteroatom hydroxylation to form Bis(2-chloroisopropyl) chloro-hydroxy-isopropyl phosphate (BCIPCHIPP), followed by nonenzymatic collapse of this intermediate to form Bis(2-chloroisopropyl) oxo-isopropyl phosphate (BCIPOIPP), and

subsequent further oxidation would result in formation of BCIPCIPP (European Chemicals Agency, 1984).

Minegishi et al. (1988) conducted a similar study on the kinetics of TCPP after a single oral administration of 16.4 mg/kg 14C-TCPP in groups of 5 Winstar rats, which were sacrificed after 3, 6, 12, 24, 72, and 168 hours and radioactivity was measured in urine, feces, expired air, blood, and various organs. After 168 h, 67.2, 22.2, 7.7 and 0.70% of the dose was recovered in urine, feces, expired air, and the carcass, respectively. In blood, the same biphasic half-life was detected and a first- and second-phase half-life of 11.7 and 58.7 hours was calculated.

In a separate experiment, the bile duct was cannulated and bile was collected following administration of 16.4 mg/kg 14C-TCPP every two hours for the first 30 hours, and from 30 till 46 hours, and 46 – 48 hours. This revealed that TCPP (and/or its metabolites) are rapidly excreted into bile following oral exposure; with 45% of TCPP being directed to bile within the first 48 hours and most of this transpiring in the first 4 hours after dosing. The authors concluded that it is likely that enterohepatic circulation of TCPP occurs, since a biliary/fecal excretion ratio of 2.23 was seen.

Both the Stauffer Chemical Company (1984) and Minegishi et al. (1988) studies determined the concentration of TCPP and/or its metabolites in different tissues by measurement of radioactivity (see Text S6-Table 1). The highest concentrations at 3 hours after exposure were detected in the liver and kidney. The relative concentration in these tissues to blood was calculated by Minegishi et al. (1988) and this declined more rapidly for the kidney than for the liver further supporting the notion that enterohepatic circulation is occurring.

Text S6-Table 1 The mean distribution (±S.E) of 14C-Radioactivity in the Blood and Tissues of Rats after Oral Administration of 14C-radiolabled TCPP in Minegishi et al. 1988 and the Stauffer Chemical report. N.D. = Not Determined

Source		(Minegishi et al., 1988)						*Staufer Chem.*	
Dose (mg/kg)		*16.4*						*20*	*200*
Time (h)		*3*	*6*	*12*	*24*	*72*	*168*	*192*	*192*
Raw Data (ng TCPP eq radiation/g tissue)	*Liver*	9382±1435	10305±570	4484±468	2621±183	1081±282	377±85	282±17	4054±370
	Kidney	8930±2450	7616±1494	3004±265	1363±26	514±16	269±36	155±13	1013±36
	Lungs	3069±495	3561±164	2349±105	852±62	488±95	193±20	159±23	1162±58
	Heart	1300±282	1445±33	593±49	445±16	236±10	72±13	135±20	798±45
	Fat/Adipose	934±229	1104±56	688±36	495±13	311±7	177±10	184±34	2072±246
	Spleen	927±102	1353±223	721±59	442±13	223±7	85±10	115±11	929±55
	Gonads / Testis	655±66	940±85	370±39	321±131	190±10	85±7	83±7	1147±197
	Muscle	603±59	868±49	570±52	334±10	216±20	115±10	71±4	761±68
	Brain	393±46	708±131	292±20	242±3	134±7	59±10	113±10	818±53
	Blood	1150±52	1513±62	672±36	478±39	242±43	85±13	N.D.	N.D.
	Small Intestine	N.D.	N.D.	N.D.	N.D.	N.D.	N.D.	80±12	2381±896
	Large Intestine	N.D.	N.D.	N.D.	N.D.	N.D.	N.D.	98±21	1338±209
	Stomach	N.D.	N.D.	N.D.	N.D.	N.D.	N.D.	75±7	780±43
	Skin	N.D.	N.D.	N.D.	N.D.	N.D.	N.D.	130±11	4950±2061
Derived C14 Radioactivity Ratios	*Liver/blood*	8.2	6.8	6.7	5.5	4.5	4.4	-	-
	Kidney/blood	7.8	5.0	4.5	2.8	2.1	3.2	-	-
	Liver / 9 Tissues	3.2	3.3	3.1	3.3	2.9	2.4	2.0	2.9
	Kidney / 9 Tissues	3.1	2.5	2.1	1.7	1.4	1.7	1.1	0.7

In addition, the NTP included toxicokinetic measurements in their toxicology studies of TCPP in rats and mice after chronic administration of TCPP (Collins et al., 2022). No consistent increasing trend in TCPP concentrations in plasma was found at increasing time points in both rats and mice, indicating an absence of bioaccumulation. After 12 months, plasma samples from male rats and female mice

were evaluated for the presence of BCIPCIPP. In both groups, BCIPCIPP concentrations were detected proportionally to the TCPP exposure, with this metabolite's concentrations 1-3 orders of magnitude higher than the TCPP concentrations in the plasma of the same animals.

Next to these in vivo studies, several experiments have been done with liver preparations. The first such study was performed by BASF (2007), public summary available in the ECHA registration dossier (European Chemicals Agency, 2007), in which TCPP was incubated in liver S9 fractions and liver slices from livers of male Wistar Han rats. S9 incubation resulted in conversion of TCPP into 58% BCIPHIPP, 20% BCIPCIPP, and 9% unchanged TCPP, while liver slice incubation resulted in 51% unchanged TCPP, 7% BCIPCIPP, 7% BCIPHIPP, and 7% of a BCIPHIPP glucuronic acid conjugate. The difference between the rate of conversion between S9 and liver slice incubation might be explained by the fact that in liver slices the three-dimensional tissue structure is largely maintained, meaning that ingress of TCPP into the tissue will be limited, but it is also plausible that a fraction of the absorbed into liver cells is excreted into the bile canaliculi.

Van den Eede et al. (2013) investigated the metabolism of TCIPP in human liver microsomes (HLM) and S9 fractions and identified BCIPP, BCIPCIPP and BCIPHIPP as metabolites with relative contributions of 20%, 30% and 40%, respectively. Abdallah et al. (2015) identified the same metabolites in addition to a Bis(2-chloroisopropyl) isopropyl phosphate –glutathione conjugate (BCIPIPP-SG) following HepG2 cell incubation.

Den Ouden et al. (2024) identified BCIPP, BCIPHIPP, BCIPCIPP, BCIPOIPP and BCIPCHIPP as TCIPP metabolites in HLMs. Based on relative areas, BCIPHIPP was the major metabolite in vitro, with BCIPOIPP identified as an intermediate product. In addition, BCIPHIPP, BCIPP, BCIPCIPP and BCIPCHIPP were identified by suspect screening in urine from the same TCPP exposed workers as used in the current study. Based on semiquantitative data, BCIPHIPP was the major metabolite present in the urine samples treated with β-glucuronidase, representing 94% of the semi-quantitatively quantified metabolites in the workers from the factory with the highest TCPP exposure (C007) (den Ouden et al., 2024). Given the sample pre-treatment with β-glucuronidase, it was not possible to state whether the BCIPHIPP was present in the urine as such or as the glucuronic acid conjugate.

To determine if it is plausible that BCIPHIPP-GlcA is present in the urine of occupationally exposed workers, several urine samples from occupationally exposed workers were extracted and analyzed with and without the pretreatment with β-glucuronidase. A clear substantial decrease in BCIPHIPP concentration was seen in all samples to which β-glucuronidase was not added compared to BCIPHIPP levels in the same samples when this enzyme was added (Text S6-Table 2). This is in line with findings reported by Bastiaensen et al. (2018) in which urine concentrations of BCIPHIPP were close to zero when β-glucuronidase was not added to the samples. These results support the view that the major metabolite in occupationally exposed humans is actually BCIPHIPP-GlcA and that β-glucuronidase is an essential step in sample preparation for the quantification of TCPP exposure.

Text S6-Table 2 Concentrations (ng) of BCIPP and BCIPHIPP in urine samples extracted with or without β-glucuronidase addition.

**conc in ng**	**BCIPP**				**BCIPHIPP**			
	1 mL		***0.5 mL***		***1 mL***		***0.5 mL***	
	Enzyme	***No enzyme***	***Enzyme***	***No enzyme***	***Enzyme***	***No enzyme***	***Enzyme***	***No enzyme***
**Sample1_1**	<LOQ	<LOQ	<LOQ	<LOQ	1.27	<LOQ	0.53	<LOQ
**Sample1_2**	<LOQ	<LOQ	<LOQ	<LOQ	1.35	<LOQ	0.56	<LOQ
**Sample2_1**	24.6	30.3	11.9	14.7	226.86	1.59	138.84	1.11
**Sample2_2**	21.2	28.3	10.8	14.9	218.91	1.53	131.11	0.79
**Sample3_1**	32.1	33.8	16.0	14.2	15.62	0.60	26.81	0.34
**Sample3_2**	30.6	22.6	15.7	14.7	13.96	0.63	33.03	0.33
**Sample4_1**	1.9	2.3	1.1	1.1	10.94	<LOQ	5.53	<LOQ
**Sample4_2**	1.9	2.1	<LOQ	1.2	11.07	<LOQ	5.44	<LOQ

The conjugation of BCIPHIPP may also explain why BCIPHIPP was not identified in the first report by the Stauffer Chemical Company (1984). The urine samples were not treated with β-glucuronidase, meaning if present most BCIPHIPP would have been conjugated. Furthermore, before introduction to GC-RAM/FID and GC-MS systems, the samples were methylated with ethanol-free etheral diazomethane, which would have added methyl groups to all available hydroxyl groups on BCIPHIPP-GlcA thus increasing the molecular weight from 485 to 541 Dalton. At such molecular weight, the retention time in gas chromatography would be substantially higher than the metabolite BCIPCIPP (20 – 21 min with the settings used) and it is doubtful if the GC-RAD/FID and GC-MS equipment at that time would have been able to detect the BCIPHIPP-GlcA metabolite.

SI-2 Tables

Table S1 Number of urine samples included in each factory and distribution of controls, foam samples and conversion samples in each factory.

Factory code	Total urine samples	Workers	Foam workers	Conversion workers	Controls
**C002**	14	6	12		2
**C006**	6	2	4		2
**C007**	14	6	8	4	2
**C012**	20	9	14	4	2
**C013**	11	5	4	6	1
**Total Urine samples**	**65**	**28**	**42**	**14**	**9**

Table S2 Instrumental parameters for the measured compounds.

Compound	IS	Precursor	Product ion	CE	FV	CAV	Polarity
**BCIPP**	BDCIPP-d10	251<br>249	35<br>35	10<br>5	166	2<br>2	Negative
**BCIPHIPP**	TCEP-d12	309<br>309	175<br>99	10<br>25	166	6<br>2	Positive

IS: internal standard, CE: collision energy, FV : fragmentor voltage, CAV: Collision cell accelerator voltage

Table S3 MS settings for analysis on the Agilent 6495 QQQ.

Parameter	Value (+ESI)	Value (-ESI)
**Dry gas temperature (˚ C)**	250	250
**Dry gas flow (L/min)**	14	14
**Nebulizer (psi)**	40	40
**Sheath gas temperature (˚ C)**	325	325
**Sheath gas flow (L/min)**	11	11
**Capillary (V)**	3000	2500
**Nozzle (V)**	0	0
**High pressure RF**	130	70
**Low pressure RF**	60	40

ESI: electrospray ionization, RF: radio frequency

Table S4 Comparison of HBGVs of TCPP in literature.

Reference	Base study used	Endpoint	Point of departure	Assessment factor	Correction oral absorption	HBGV (ng/kg bw/day)
**Current Study**	**NTP, 2023 (chronic part)**	**Hepatocellular adenoma or carcinoma**	**NOAEL of 329 mg/kg bw/day**	**17.5 interspecies<br>5 for intraspecies for workers<br>10 for intraspecies for consumers**	**0.8**	**1 500 000 (general population)<br>3 000 000 (worker)**
(EU RAR, 2008)	Stauffer Chemical Company, 1981	Increased absolute and relative liver weight and mild thyroid follicular cell hyperplasia	LOAEL of 52 mg/kg bw/day	10 for interspecies<br>5 for intraspecies in workers<br>10 for intraspecies in consumer	0.8	420 000 (general population)<br>840 000 (worker)
(Van den Eede et al., 2011)	Stauffer Chemical company, 1981	Changes in liver weight and cellular changes in kidney	NOAEL of 52 mg/kg bw/dy	10 000 in line with earlier work (Hartmann et al., 2004; Saito et al., 2007)	Not applied	8 000
ECHA screening report (European Chemicals Agency, 2018)	Stauffer Chemical Company, 1981	Increased absolute and relative liver weight and mild thyroid follicular cell hyperplasia	LOAEL of 52 mg/kg bw/day	10 for interspecies<br>5 for intraspecies in workers<br>10 for intraspecies in consumer<br>6 for dose-relationship<br>2 for extrapolation from subchronic to chronic	0.8	35 000 (general population)<br>70 000 (worker)
(Zhao et al., 2019)	Not mentioned	not mentioned	Not mentioned	NOAEL/1000	Not mentioned	3600
(US EPA, 2023)	NTP, 2023 (subhronic part)	Hepatocyte hypertrophy	$BMDL_{10}$ of 138 mg/kg bw/day	10 for interspecies<br>10 for intraspecies<br>10 for lack of developmental studies in database<br>10 for extrapolation from subchronic to chronic	Not applied	10 000

SI-3 Figures

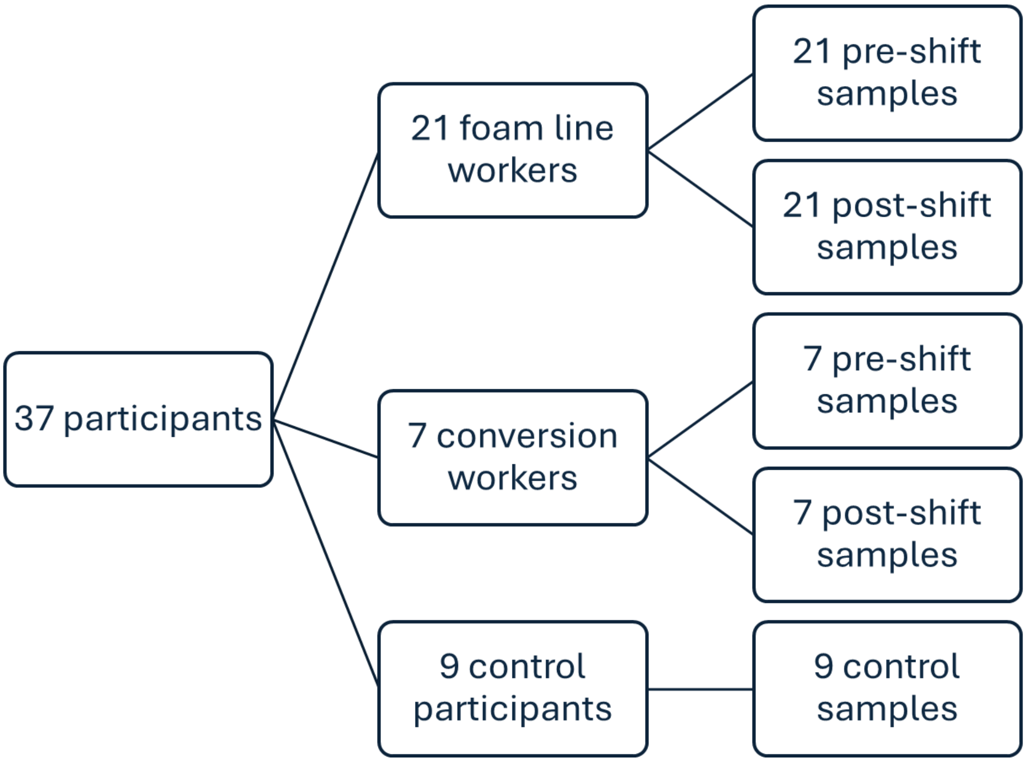

Figure S1 Overview of study participants, their function and urine samples provided.

References

Abdallah MAE, Zhang J, Pawar G, Viant MR, Chipman JK, D'Silva K, Bromirski M, & Harrad S. High-resolution mass spectrometry provides novel insights into products of human metabolism of organophosphate and brominated flame retardants. *Analytical and Bioanalytical Chemistry*, 2015: *407*(7), 1871–1883. https://doi.org/10.1007/s00216-015-8466-z

Bastiaensen M, Xu F, Been F, Van den Eede N, & Covaci A. Simultaneous determination of 14 urinary biomarkers of exposure to organophosphate flame retardants and plasticizers by LC-MS/MS. *Analytical and Bioanalytical Chemistry* 2018: *410*(30), 7871–7880. https://doi.org/10.1007/s00216-018-1402-2

Collins B, Slade D, Aillon K, Stout M, Betz L, Waidyanatha S, & Ryan K. Plasma concentrations of tris(1-chloro-2-propyl) phosphate and a metabolite bis(2-chloroisopropyl) 1-carboxyethyl phosphate in Sprague-Dawley rats and B6C3F1/N mice from a chronic study of tris(chloropropyl) phosphate via feed. *Toxicology Reports*, 2022: *9*(March), 690–698. https://doi.org/10.1016/j.toxrep.2022.03.025

The Danish Environmental Protection Agency (2014). *Survey of Tris (2-chloro-1-methylethyl)-phosphate* (Issue October). https://www2.mst.dk/Udgiv/publications/2014/01/978-87-93026-96-4.pdf

de Kort, P. (2023). *SAFETY GUIDELINES FOR THE FLEXIBLE POLYURETHANE FOAM INDUSTRY* (Issue September). https://europur.org/wp-content/uploads/2023/09/Safety-Guidelines-2023-1.pdf

den Ouden F, Estévez-Danta A, Belova L, Gys C, Klimowska A, Roggeman M, Van Wichelen N, Quintana JB, Rodil R, Poma G, & Covaci A. Investigation of in vitro biotransformation of tris (1-chloro-2-propyl) phosphate and confirmation in human urine. *Current Research in Toxicology*, 2024: *6*(December 2023). https://doi.org/10.1016/j.crtox.2024.100164

EU RAR. (2008). *European Union Risk Assessment Report Tris(2-chloro-1-methylethyl) phosphate (TCPP).* https://echa.europa.eu/documents/10162/13630/trd_rar_ireland_tccp_en.pdf/315063b0-593d-4703-9519-562c258506e6

European Chemicals Agency. (1984). Reaction products of phosphoryl trichloride and 2-methyloxirane. https://echa.europa.eu/registration-dossier/-/registered-dossier/27081/7/2/2/?documentUUID=d8838b52-3828-4b84-8a2e-332aa0c82c50

European Chemicals Agency. (2007). Reaction products of phosphoryl trichloride and 2-methyloxirane. https://echa.europa.eu/registration-dossier/-/registered-dossier/27081/7/2/2/?documentUUID=e4af59f2-cbe0-4be6-84a8-2c37482edd7d

European Chemicals Agency. (2012). Guidance on information requirements and chemical safety assessment. Chapter R.8: Characterisation of dose [concentration] - response for human health. Guidance on Information Requirements and Chemical Safety Assessment, November, 1–186. https://echa.europa.eu/documents/10162/17224/information_requirements_r8_en.pdf

European Chemicals Agency. (2015). Guidance on information requirements and chemical safety assessment : Chapter R.12 : use description, version 3.0 (Issue December). https://echa.europa.eu/documents/10162/17224/information_requirements_r12_en.pdf/ea8fa5a6-6ba1-47f4-9e47-c7216e180197

European Chemicals Agency. (2017). REACH Registration Dossier: Reaction products of phosphoryl trichloride and 2-methyloxirane-7.8.2. Developmental toxicity/teratogenicity-002 Key Experimantal Study. https://chem.echa.europa.eu/100.235.857/dossier-view/fcdbfb2b-9ee0-

44d2-b347-f10121b4e478/c7054035-e26e-42f6-bbaa-e8eec853da23_929d879c-5164-4c71-9026-c8ebdd04209f?searchText=tcpp

European Chemicals Agency. (2018). *Screening Report. An assessment of whether the use of TCEP, TCPP and TDCP in articles should be restricted*. *April*, 1–68. https://echa.europa.eu/documents/10162/13641/screening_report_tcep_tcpp_td-cp_en.pdf/e0960aa7-f703-499c-24ff-fba627060698

Hartmann PC, Bürgi D, & Giger W. Organophosphate flame retardants and plasticizers in indoor air. *Chemosphere*, 2004: *57*(8), 781–787. https://doi.org/10.1016/j.chemosphere.2004.08.051

Klaunig JE, Dekant W, Plotzke K, & Scialli, AR. Biological relevance of decamethylcyclopentasiloxane (D5) induced rat uterine endometrial adenocarcinoma tumorigenesis: Mode of action and relevance to humans. *Regulatory Toxicology and Pharmacology*, 2016:*74*, S44–S56. https://doi.org/10.1016/j.yrtph.2015.06.021

Maronpor, R. R. (n.d.). *NTP Nonneoplastic Lesion Atlas-Liver-[Basophilic, Eosinophilic, Clear Cell, Mixed] Focus*. Retrieved January 6, 2025, from https://ntp.niehs.nih.gov/atlas/nnl

Minegishi KI, Kurebayashi H, Nambaru S, Morimoto K, Takahashi T, & Yamaha T. Comparative Studies on Absorption, Distribution and Excretion of Flame Retardants Halogenated Alkyl Phosphate in Rats. *Eisei Kagaku*, 1988: *34*, 102–114.

National Institute for Public Health and the Environment. (2024). PROAST. https://www.rivm.nl/en/proast

NTP. (2020). NTP Developmental and Reproductive Toxicity Technical Report on the Prenatal Development Studies of Tris(choropropyl)phosphate in Sprague Dawley Rats. 6.

NTP. (2023). *NTP Technical Report on the Toxicology and Carcinogensis Studies of an Isometic Mixture of Tris (chloropropyl) phosphate Adminstered in Feed to Sprague Dawley Rats and B6C3F1/N Mice*.

Saito I, Onuki A, & Seto H. Indoor organophosphate and polybrominated flame retardants in Tokyo. *Indoor Air*, 2007:*17*(1), 28–36. https://doi.org/10.1111/j.1600-0668.2006.00442.x

Stauffer Chemical Company. (1981). Fyrol PCF 3-month dietary sub-chronic toxicity study in rats.

Svara J, Weferling N, & Hofmann T. Phosphorus Compounds, Organic. In *Ullmann's Encyclopedia of Industrial Chemistry* 2006 (pp. 19–46). Wiley. https://doi.org/10.1002/14356007.a19_545

Truong JW, Diamond ML, Helm PA, Jantunen LM. Isomers of tris(chloropropyl) phosphate (TCPP) in technical mixtures and environmental samples. Analytical and Bioanalytical Chemistry, 2017:*409*, 6989-6997.

United States Environmental Protection Agency. (2012). Provisional Peer-Reviewed Toxicity Values for Tris(1-chloro-2-propyl)phosphate. https://hhpprtv.ornl.gov/issue_papers/Tris1chloro2propylphosphate.pdf

United Stated Environmental Protection Agency. (2023). *Regional Screening Level (RSL) Subchronic Toxicity Supporting Table May 2023*. *30560*. https://semspub.epa.gov/work/HQ/404091.pdf

Van den Eede N, Dirtu AC, Neels H, & Covaci A. Analytical developments and preliminary assessment of human exposure to organophosphate flame retardants from indoor dust. *Environment International*, 2011:*37*(2), 454–461. https://doi.org/10.1016/j.envint.2010.11.010

Van den Eede, N, Heffernan AL, Aylward LL, Hobson P, Neels H, Mueller JF, & Covaci A. Age as a determinant of phosphate flame retardant exposure of the Australian population and

identification of novel urinary PFR metabolites. *Environment International*, 2015:*74*, 1–8. https://doi.org/10.1016/j.envint.2014.09.005

Van den Eede N, Maho W, Erratico C, Neels H, & Covaci A. First insights in the metabolism of phosphate flame retardants and plasticizers using human liver fractions. *Toxicology Letters*,2013:*223*(1), 9–15. https://doi.org/10.1016/j.toxlet.2013.08.012

Van den Eede N, Tomy G, Tao F, Halldorson T, Harrad S, Neels H, & Covaci A. Kinetics of tris (1-chloro-2-propyl) phosphate (TCIPP) metabolism in human liver microsomes and serum. *Chemosphere*, 2016:*144*, 1299–1305. https://doi.org/10.1016/j.chemosphere.2015.09.049

Wikoff DS, Rager JE, Haws LC, & Borghoff, SJ. A high dose mode of action for tetrabromobisphenol A-induced uterine adenocarcinomas in Wistar Han rats: A critical evaluation of key events in an adverse outcome pathway framework. *Regulatory Toxicology and Pharmacology*, 2016:*77*, 143–159. https://doi.org/10.1016/j.yrtph.2016.01.018

Zhao L, Jian K, Su H, Zhang Y, Li J, Letcher RJ, & Su G. Organophosphate esters (OPEs)in Chinese foodstuffs: Dietary intake estimation via a market basket method, and suspect screening using high-resolution mass spectrometry. *Environment International*, 2019:*128*(April), 343–352. https://doi.org/10.1016/j.envint.2019.04.055
